# A hybrid RAG and rule-based reasoning framework for technical document analysis

**DOI:** 10.3389/frai.2026.1922508

**Published:** 2026-09-17

**Authors:** Esther Rachel Thomas, Niranchna Natarajan, Keerthana Jayaprakashan, A. Malini

**Affiliations:** School of Computer Science and Engineering, Vellore Institute of Technology, Chennai, India

**Keywords:** deterministic retrieval, geometric spatial indexing, knowledge graph, retrieval-augmented generation, rule extraction, safety-critical systems, SAT-based inference

## Abstract

The ability to access specific operational guidelines in unstructured technical documentation is a crucial and unsolved problem in safety sensitive areas like aerospace, clinical care, and regulatory compliance, where approximate retrieval and hallucination are unacceptable. Current systems of Retrieval Augmented Generation (RAG) relying on dense vector embeddings are unable to offer the deterministic accuracy such environments require. In this paper, a neurosymbolic framework is provided to overcome this limitation through the translation of natural language documents into three complementary representations of symbolic knowledge: a SQLite (Lightweight SQL Database Engine) database of SAT (Boolean Satisfiability) - based propositional reasoning Conjunctive Normal Form (CNF) clauses, a directed property graph relational traversal and a geometric spatial index which represents the quantitative conditional rules as axis aligned hyper rectangles in an eight dimensional parameter space. Each of the three representations is built on the same LLM (Large Language Model) - based rule extraction pipeline at the same time during offline stage, where there is no embedding of vectors of any type. Online three path reasoning architecture combines SAT at query time entailment checking, knowledge graph traversal, and exact geometric containment testing — translating the operational state of a user to a parameter space point and recalling all rules with hyper rectangular regions containing the point. The outputs of all the three paths are combined before constrained answer generation, basing each answer on logical, relational, and numerical evidence. Because retrieval is performed using mathematically precise symbolic procedures rather than similarity approximation, the SAT, graph, and geometric reasoning components are deterministic and fully explainable; large language model calls are still used for rule extraction and answer synthesis, so the system as a whole is hybrid rather than fully deterministic, and a faithfulness filter together with context-confined generation substantially mitigate, rather than formally guarantee, hallucination. The experimental findings show that this tri-representation strategy markedly enhances retrieval accuracy and reliability compared to embedding-based baselines in complicated operational query conditions, developing a scalable base of rule-based decision support in high stakes areas.

## 1 Introduction

The exponential growth of digital documentation in various technical, procedural and regulatory areas poses significant challenges in knowledge access, reasoning and interpretation. Today’s organizations keep volumes of documents containing essential rules, constraints, dependencies, and knowledge of the domain that require a high level of accuracy in interpretation. Such information is to be used efficiently for proper decision-making, compliance checks, and reliable reasoning about operations.

Conventional document management and information retrieval systems use keyword matching or semantic similarity, which are unsuitable for structured reasoning, traceability and logical verification. The approaches rarely consider the relationships and constraints hidden in documentation. There is need for intelligent systems to reason on the increasing scale and complexity of such data in a deterministic, explainable and auditable manner.

This paper addresses these challenges through proposing a framework to turn document content into structured knowledge that machines can understand. Using formal reasoning over extracted knowledge, the proposed approach can improve accuracy, trustworthiness, and clarity of information retrieval under complex and high-risk situations.

### 1.1 Overview

This project presents a framework that aims to make knowledge retrieval from unstructured technical documents precise, explainable, and deterministic. The main objective is to bridge the gap between natural language documentation and formal reasoning systems by converting textual contents into structured representations which can support logical inference, relational reasoning, and numerical validation. There are two main phases of the system; an offline knowledge construction phase and an online query processing phase. Specifically, the system functions over two phases - knowledge construction performed offline and query processing done online. These portions are then transformed into structured rule objects using a large language model (LLM). The rules embody different types of domain knowledge such as conditional relationship, constraints, procedures, and exclusions. These rules are then transformed and stored under three mutually reinforcing knowledge representations, these are SAT-based reasoning over a relational database of Conjunctive Normal Form (CNF) clauses, relational traversal utilizing a directed knowledge graph, and geometric spatial index representation encoding quantitative rules as hyper-rectangular regions within a multi-dimensional parameter space. In the online phase, user natural language queries are fed into a multi-path reasoning architecture. First, relevant parameters, propositions, or numerical values are identified based on the query. Then, the query is checked with SAT-based entailment for logical reasoning, knowledge graph traversal for relational reasoning, and geometrical containment in the parameter space for numerical reasoning in three reasoning paths in parallel. This context is then used for producing the final answer as a brief natural language statement. This project’s key contribution is that it combines several symbolic representation aspects in one framework, removing the use of probabilistic vector embedding, thus supporting deterministic retrieval. Every system response is logically explainable by referring back to clauses, relations, or geometrical conditions presented in the original documents or the resulting data structures. LLM based extraction combined with formal reasoning techniques offers a scalable and reliable approach to knowledge retrieval in complicated and safety critical domains.

### 1.2 Literature review

Extracting specific, practical rules out of large amounts of unstructured technical literature is among the more persistent open problems in safety critical systems such as aerospace, medical care, and regulatory compliance. In these settings, decisions must be based on clearly defined operational limits and verifiable rules. There is little room for ambiguity, and even small retrieval errors can lead to serious consequences. The difficulty, however, is not merely in locating relevant information, but in locating it with deterministic accuracy, a feature that the existing retrieval systems cannot offer.

Since Lewis et al. (2020) introduced the Retrieval Augmented Generation (RAG) framework, the field has experienced a significant change, confirming the value of both the parametric knowledge contained in Large Language Models (LLMs) and the use of external document retrieval when it comes to answering knowledge-intensive questions without having to retrain. The concept was brought to the public’s attention. By 2024, the community had become familiar with the Naive, Advanced and Modular approaches that Arslan et al. (2024) and Gao et al. (2024) looked at in detail. Nevertheless, as these systems became more complex, their inability to fail could no longer be overlooked. Even the retrieval of a small number of irrelevant documents can have a major effect on factual grounding (Cuconasu et al., 2024), and the phenomenon of word-level hallucination in RAG outputs as demonstrated in (Niu et al., 2024) is a natural result of the approximate search based on semantic similarity, a process which is likely to lead to confabulation. The proposed solutions, RAG Fusion (Rackauckas, 2024) and context reordering (Jin et al., 2024), do offer real improvements and yet still operate within the embedding paradigm, meaning that the approximation will continue to exist.

More sophisticated projects have since focused on architectural complexity. For example, systems such as ComoRAG (Wang et al., 2025) and SitEmb-v1.5 (Wu et al., 2025) keep a record of the reasoning process and maintain global context in order to ensure coherence across documents; multimodal pipelines have incorporated layout-aware chunking (Tripathi et al., 2025) so as to take into account the structure of the documents; and domain-specific systems have stacked a number of reasoning steps on one another in order to make the results more specific. More directly related to the present approach, several recent systems combine retrieval with explicit symbolic or logical components: SymRAG (Hakim et al., 2025) adaptively routes queries between neural and symbolic retrieval, LCR-RAG (Zheng et al., 2026) applies neuro-symbolic reinforcement learning to improve logical consistency, and other work explores neurosymbolic retrievers (Saxena and Gaur, 2026) and multi-stage retrieval-augmented generation architectures for context relevance (Aytar et al., 2025; Pichai, 2023). These are significant advances, but all the knowledge is still encoded in the form of dense vector embeddings (Karpukhin et al., 2020; Guu et al., 2020; Chen et al., 2017) and is retrieved using nearest neighbor classification. This design choice represents an irreducible approximation and is not a difficult one to make since it is necessary to have rules that must be remembered exactly. This assessment is supported by the various problems relating to data integrity. Knowledge integrity is an important area of research in retrieval systems (Mu et al., 2026) and file type identification is a broader principle according to which reliable systems should be based on verifiable and inspectable representations, not on opaque approximations (Karampidis et al., 2017; Amirani et al., 2010).

All of these threads end up identifying the same gap—namely, the need for retrieval systems that guarantee correctness without relying on similarity-based approximation. The present paper proposes a framework to address that gap. Instead of storing knowledge in the form of embeddings, the system translates unstructured natural language documents into three complementary symbolic representations, thereby enabling accurate, deterministic, and fully interpretable query answering.

The process occurs in two stages. In the offline stage, the documents are split into fragments that are semantically coherent (Beltagy et al., 2020) and are labeled by an LLM in order to identify the material that expresses rules. This information is then passed on to a structured extraction pipeline which produces typed rule objects—namely, if-then implications, constraint bounds and ordered procedural steps—and at the same time stores them in three different knowledge repositories: a relational database containing clauses in Conjunctive Normal Form (CNF) for use with SAT solving in order to carry out propositional reasoning; a directed property graph for use in relational traversal and interpretation; and a geometric spatial index which encodes quantitative conditional rules as axis-aligned hyperrectangles in an eight-dimensional parameter space. During the online stage, queries are solved by three parallel reasoning directions, including SAT-based logical entailment, graph traversal, and geometric containment testing.

The key architectural intuition is the retrieval primitive of quantitative rules that is based on precise geometric containment. A given operational state is coded as a point in the parameter space; the applicability of a rule is tested by checking whether the point lies within the associated hyperrectangle. It is a mathematically precise operation — there is no approximation and no confabulation risk of the embedding-based retrieval. Most importantly, all three knowledge stores do not make use of any type of vector embeddings. Everything is stored symbolically, thus the system is interpretable, auditable, and completely deterministic.

This work has three fold contributions. To begin with, a two-phase neurosymbolic pipeline which transforms unstructured technical documents into three concurrently built symbolic representations of knowledge. Second, a geometric spatial indexing scheme, which represents conditional rules as axis aligned hyperrectangles and provides accurate point in region retrieval with sub-milliseconds latency. Third, a parallel reasoning architecture with three paths that combines SAT propositional inference, directed graph traversal, and geometric spatial look up into a single evidence-based query processing framework (Yao et al., 2023).

### 1.3 Scope

The system is best suited to domains where source documents contain explicit conditional statements, rules tend to be primarily quantitative thresholds, the domain is reasonably stable, and queries are about particular values of the parameters. These seven domains, representing the following systems: traffic management, hospital patient care, rocket engine operations, bank loan processing, inventory management, access control and temperature control, are representative of this scope.

The choice of the 8-dimensional parameter space is not a design limitation, it is a configuration. The geometric path can be used to any domain that contains quantitative threshold rules without changing the geometric encoding or containment testing parts so that the dimension registry can be extended to any parameter set in that domain. Likewise, the limitation of only processing plain text documents can be overcome by having a preprocessing layer, which can be implemented through the ‘pdfplumber’ or ‘python-docx’ libraries, before the reasoning process is applied to the chunks.

The immediate use of this framework is the automation of the encoding of rules in safety-critical onboard decision systems, which is the major application and is the process with which the operational documents are now translated into certified rule sets, typically taking many months of manual work when performed by one or a few experts. This directly tackles the “motivation” of this work, namely that safety-critical systems already rely on a deterministic rule evaluation, and that the key problem to address is to automate the knowledge construction phase that is a limiting factor in the speed and scalability of using these systems in practice.

### 1.4 Novelty and positioning relative to prior art

Representing numeric rules as axis-aligned hyperrectangles and retrieving by containment is not, by itself, a new idea: it traces to Salzberg’s Nested Generalized Exemplars (hyperrectangle-based) learning and the broader hyperrectangle machine-learning literature, and resembles patent literature on automatic refinement of hyperregion rulesets from labeled data. SAT solving, knowledge-graph traversal, and retrieval-augmented generation are each independently well-established techniques, and this should be stated plainly: claiming the geometric representation itself as the contribution would not withstand scrutiny against this prior art.

What this work adds, scoped to what has actually been demonstrated, is threefold. First, an LLM-driven pipeline that automatically derives hyperrectangle regions -- and the accompanying SAT and graph representations -- from raw, unstructured natural-language prose rather than from pre-labeled numeric training data as the hyperrectangle literature assumes; this was verified on genuinely varied real-world phrasing (direct statements, modal phrasing, negated constructions, passive constructions, multi-sentence causal chains), not only clean templated text Second, through a single shared extraction process, tri-path integration is achieved by assigning one identifier to each rule, which ensures that all three stores remain consistent by design rather than after the fact. Third, a division of labor between the three paths that has been determined on the basis of empirical evidence rather than being assumed — the reasoning-path ablation study (Section 4.4.2) and an independent comparison-harness experiment (Section 4.4.4) both demonstrate that SAT + Graph matches the full three-path system when assessed using aggregate correctness metrics, although a breakdown by category shows that Geometric is the only path which correctly excludes queries whose numbers do not actually satisfy any rule. The more precise and well-supported claim is thus that geometric containment functions as a precision mechanism specifically for numeric and refusal-type queries, and not as an additional source of coverage — this is a more limited claim than “three complementary paths,” but one that is actually supported by the aggregate data.

## 2 System architecture

### 2.1 Offline phase: document ingestion and knowledge construction

#### 2.1.1 Overview

The first stage of the process is the offline stage, during which unstructured natural language technical documents are transformed into structured formats that can be queried by machines. The input materials—most often written in plain English as policies, procedures or technical specifications—are read and then passed through a single rule extraction pipeline, which deals with all the documents together. Section 2 (System Architecture) explains what the system is — that is, its various structural components and the way they are connected: the offline knowledge-construction pipeline, the three reasoning stores, and the online query pipeline. Section 3 (Methodology) then deals separately with the PROCESS involved in building and evaluating those components — including the design choices made regarding the extraction prompts, the encoding schemes, and the evaluation protocol. The two sections are intentionally kept separate: architecture provides a static description of the system’s components, whereas methodology refers to the procedures used in constructing, extracting information from, and testing those components; Section 3 assumes the architecture outlined here and does not repeat it (Figure 1).

**Figure 1:**
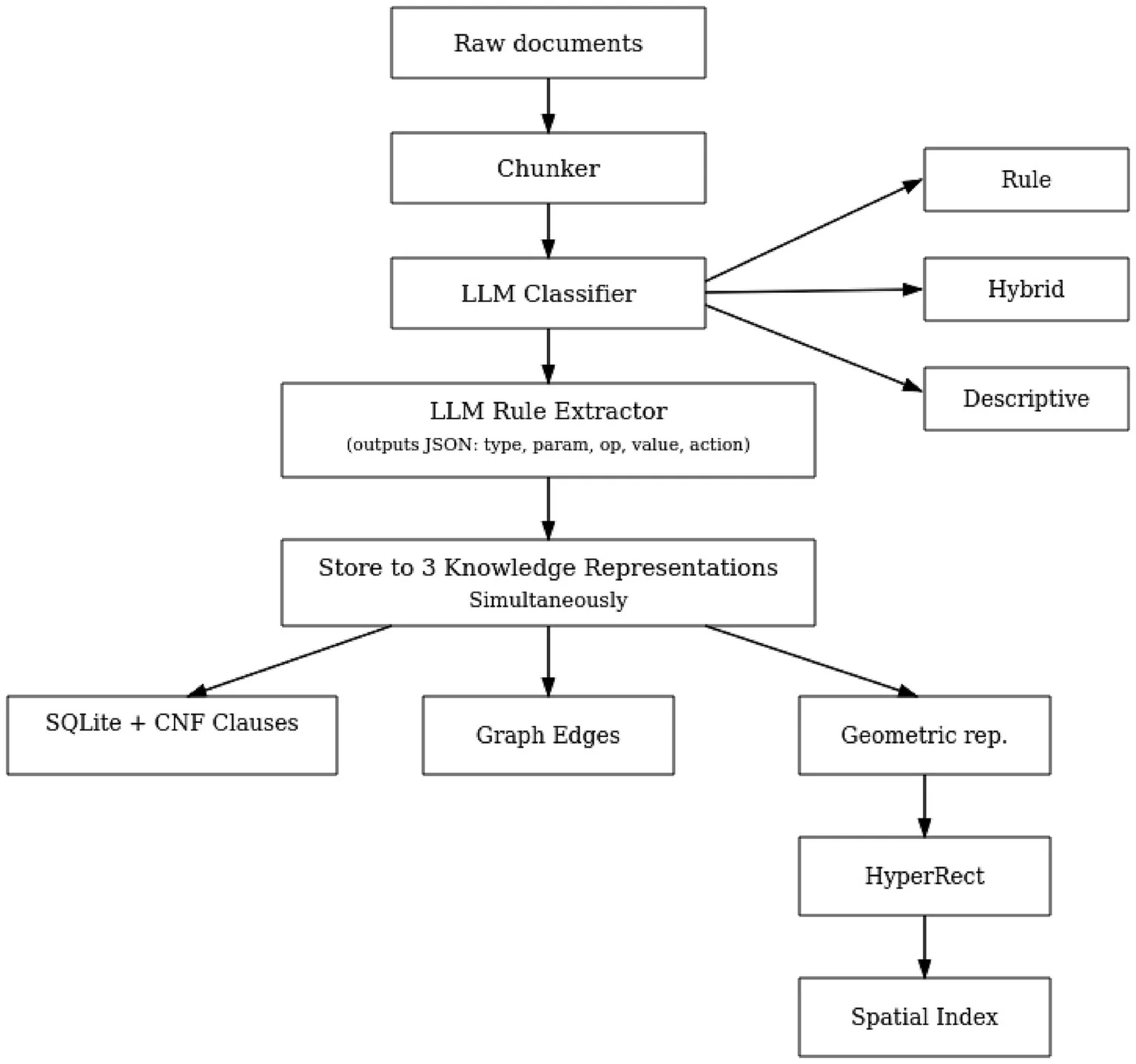
Offline phase architecture of the proposed system.

From this one extraction process the system will create three different knowledge stores, each of which is optimized for different modes of computation and reasoning. The first one is a relational database of propositional logic clauses in Conjunctive Normal Form (CNF), which makes it easy to solve satisfiability (SAT) problems with logical constraints. The second one is a directed property graph which is constructed with help of NetworkX that lets us move through entities and their relationships in a structured form. The third is a spatial index that consists of hyperrectangles, which are intended to answer geometric containment queries on numerical or range constrained queries.

Most importantly, the three representations are based on the same extracted rules, which provides consistency between modalities of reasoning. When the offline stage is finished, the knowledge that was originally a set of structures human readable in prose is made accessible to the system in the form of a set of complementary, milliseconds queryable structures — each of which is optimally adapted to a particular type of inference problem.

#### 2.1.2 Module description

##### 2.1.2.1 Document loading

Raw documents are in plain text format and they are stored in a special input directory. The system scans files one by one and stores the entire text content in memory. No processing or formatting is done at this stage. The system is document agnostic — it does not need domain specific configuration or schema definition before processing.

##### 2.1.2.2 Sentence aware chunking

A sentence aware splitting strategy is an important factor in rule integrity. An unsophisticated character-based division has the danger of cutting a rule at a random point, with the condition clause in one part and the action clause in the other. Sentence aware splitting makes sure that all the conditional rules are not lost in one chunk.

Concretely, text is split only at sentence boundaries (.!?), and a running chunk is closed once it approaches a target of 1,200 characters, with the next sentence starting a new chunk; a single sentence that itself exceeds 1,200 characters is kept whole as its own oversized chunk rather than being split, since a guaranteed-intact rule is preferable to a guaranteed-broken one. The 1,200-character target is an empirical choice suited to a 3B-parameter local model (llama3.2) running on CPU (Central Processing Unit) hardware, balancing per-call latency against the risk of the model losing track of content in an overly long prompt, and is a tunable parameter rather than a fixed constant. A period-based sentence boundary is additionally required to be followed by an uppercase letter or the end of the text before it is treated as a real sentence break, after testing found that naive period-splitting was fooled by abbreviations and decimal numbers (e.g., “Dr.,” “e.g.,” “3.5 bar”), incorrectly treating them as chunk boundaries.

###### 2.1.2.2.1 Worked example

Given the input “Patient discharge planning is a complex process that begins at admission. The clinical team coordinates to ensure safe transitions of care. If a patient’s systolic blood pressure remains above 160 mmHg, the patient should not be cleared for discharge without a specialist cardiology review,” the chunker produces a single chunk containing all three sentences: the rule-bearing final sentence stays fully intact alongside its preceding background sentences, confirming the sentence-aware boundary is respected rather than truncating the rule mid-condition.

##### 2.1.2.3 LLM based classification

The classifier returns one of three labels. A label of rule indicates the chunk contains explicit conditional logic and should proceed to full extraction. A label of hybrid indicates a mixture of rule bearing and descriptive content. A label of descriptive indicates background information containing no actionable rules.

The classification acts as an efficiency filter. Technical documents usually consists substantial quantities of contextual, historical and descriptive content which carries no logical and operational rules. The classification step ensures that this expensive extraction LLM call is made only for the content that results in structured rules.

If the classification output is either ambiguous or the classification call itself fails, the chunk is labeled hybrid rather than descriptive and still proceeds to extraction. An earlier version of this fallback defaulted to descriptive, which testing found would silently drop real rules whenever the classification call failed or returned an unparseable label; the corrected fallback reflects the judgment that a rule which is silently never extracted is a more serious failure than a purely descriptive chunk that is extracted and simply yields nothing at the extraction stage.

##### 2.1.2.4 Structured rule extraction using LLM

This part of the model is what requests the LLM with a prompt that has been structurally extracted, the result of which is a list of rule objects in JSON format. The three kinds of rules are: if-then rules which express a casual implication; constrain rules which indicate absolute restrictions on measurable parameters and procedural rules which express a sequence of steps to be followed in order.

For example, the sentence ‘If a patient’s blood pressure’s above 160 systolic specialist review is required’ is considered to have a rule that we need to follow. This rule is written in a format that a computer can understand: it says what kind of rule it is, what blood pressure measurement it is talking about that the measurement is too high what the high measurement is and what we need to do about it. The rule is about the patient’s blood pressure. The patient’s blood pressure is the thing here. We need to check the patient’s blood pressure to see if it is too high. On the hand the sentence ‘The discharge procedure requires: confirm vital signs, reconcile medications, schedule follow-up review instructions obtain physician sign-off’ is a list of things to do. This list of things to do is a procedure. The procedure has steps. We need to do these steps in order. The procedure is, about what to do when a patient is being discharged. The discharge procedure is important. The discharge procedure has parts. The list of things to do is given in a format that a computer can understand. This format is helpful because it tells the computer what to do. The computer does not need to try to understand what the words mean. The computer can just follow the instructions.

An honest limitation was identified directly. When a real run was carried out on a double-negative source sentence—that is, “the patient should not be cleared for discharge without a specialist cardiology review”—it yielded one correct if-then rule together with a second, incorrect constraint rule in which the action was the opposite of that in the source (“clear for discharge” rather than blocking discharge). This result was found to be reproducible in two independent runs using the same input and is presented as a true, citable limitation concerning the handling of negation in the current extraction prompt, rather than being attributable to the faithfulness filter’s word-overlap check, which alone cannot detect an inverted action that still uses the same vocabulary as the source.

##### 2.1.2.5 Faithfulness verification

###### 2.1.2.5.1 Example prompts (reproducibility)

The short version of the classification prompt is: “If the text has any logic a limit or a direction (like ‘if X then Y’ or ‘X should be less than Y’) mark it as ‘rule’. It should be marked ‘descriptive’ only if it has background or past details with no conditional logic; if it has both kinds of content then mark it ‘hybrid’. [Here are three examples to show this.] Your answer needs to be one word “The short extraction prompt is: “Find all the rules in the text below and show them as a JSON list. Every entry in the list needs a ‘type’ field, which can be ‘if-then’ ‘constraint’ or ‘procedure’ [the complete format for multiple-condition rules is given, along with an example that is made up on purpose rather than real because a previous real example was copied back by the model instead of having the rules taken from the real text]. Only the JSON list should be returned.” Full versions of both prompts are, in the released extraction code to make sure it can be done the same way.

The rules which have been extracted are verified against the source chunk from which they were supposed to have been drawn; the important words in the claimed action, condition, or in the procedure steps of the rule are compared with those in the source text, and the rule is rejected if the degree of overlap is below a predetermined threshold. This procedure picks up cases of hallucination that occur during the extraction process -- the situation where the LLM comes up with a rule that sounds plausible but is not actually supported by the source text -- before it is included in the knowledge base. This is a heuristic safeguard and not a formal guarantee of faithfulness.

##### 2.1.2.6 Confidence score assignment

This heuristic serves as a stand-in for extraction quality and is employed at query time to rank the matched rules from the highest to the lowest confidence.

##### 2.1.2.7 Knowledge store construction

The rules that are extracted are applied simultaneously to all three of the knowledge representations, which are developed at the same time using the same rule object, thus ensuring consistency throughout all the reasoning paths.

Path 1: SQLite database and CNF clauses (SAT reasoning). The rule-to-clauses() function carries out the transformation of each rule into a clause in conjunctive normal form; for if-then rules the material implication P → Q is converted into its logically equivalent disjunctive form, namely ¬P ∨ Q; a single positive unit clause is created for constraint rules; for procedure rules the unit clauses are connected by ordering implications which take the form of ¬step_i ∨ step_i + 1; the SQLite database is in charge of keeping track of all the rules and includes records containing the rule type, a JSON representation, a confidence score, the source filename, and an MD5 (Message Digest Algorithm 5) hash to ensure that there are no duplicates.

Path 2: Knowledge Graph (cf. Ji et al., 2022; Pan et al., 2024). Nodes represent entities which are parameter names, condition labels, action descriptions, and procedure steps. Edges are responsible for representing typed relations. In order to record the Unix time of extraction, ever edge carries a source attribute and a timestamp attribute.

Path 3: Geometric Spatial Index. This block transforms each rule dict into one or more HyperRect objects representing the rule as an axis aligned region in the 8 dimensional parameter space. For conjunctive (AND) rules, bounds are intersected per dimension producing a single hyperrectangle. For disjunctive (OR) rules, each condition independently produces a separate HyperRect.

#### 2.1.3 Output of offline phase execution

At the end of the offline phase, the system has generated three fully populated knowledge stores which collectively encode the entire operational knowledge of the corpus. The full rule inventory in CNF clauses and the propositions inverted index are stored in the SQLite database. The directed NetworkX graph represents the relational structure. The spatial index represents all quantitative conditional rules as geometric regions allowing retrieval at sub millisecond latency. Notably, none of these three representations use any vector embeddings — everything is represented symbolically, making the reasoning components interpretable, deterministic and auditable. The system as a whole is hybrid because LLM calls are used for rule extraction and answer synthesis (Table 1), but all symbolic reasoning is deterministic.

**Table 1:** Deterministic vs. non-deterministic components of the system.

Deterministic components (no LLM)	Non-deterministic components (LLM calls)
SAT-based entailment checking (proof by refutation)	Rule extraction from raw text (classification + extraction)
Knowledge graph construction and traversal	Candidate proposition matching (SAT semantic-bridging fallback, used only when deterministic keyword/synonym matching fails)
Hyperrectangle containment testing	Final answer synthesis (constrained to retrieved context)
Keyword/regex-based query-to-point extraction	—

### 2.2 Online phase: query processing

The online stage is responsible for converting a user query into a grounded response that is based on precomputed knowledge structures. It consists of four steps: keyword extraction, parallel retrieval, context merging, and constrained answer generation. The three parallel reasoning directions are SAT-based logic reasoning, knowledge graph traversal and geometric spatial look up access complementary evidence in different knowledge representations (Figure 2).

**Figure 2:**
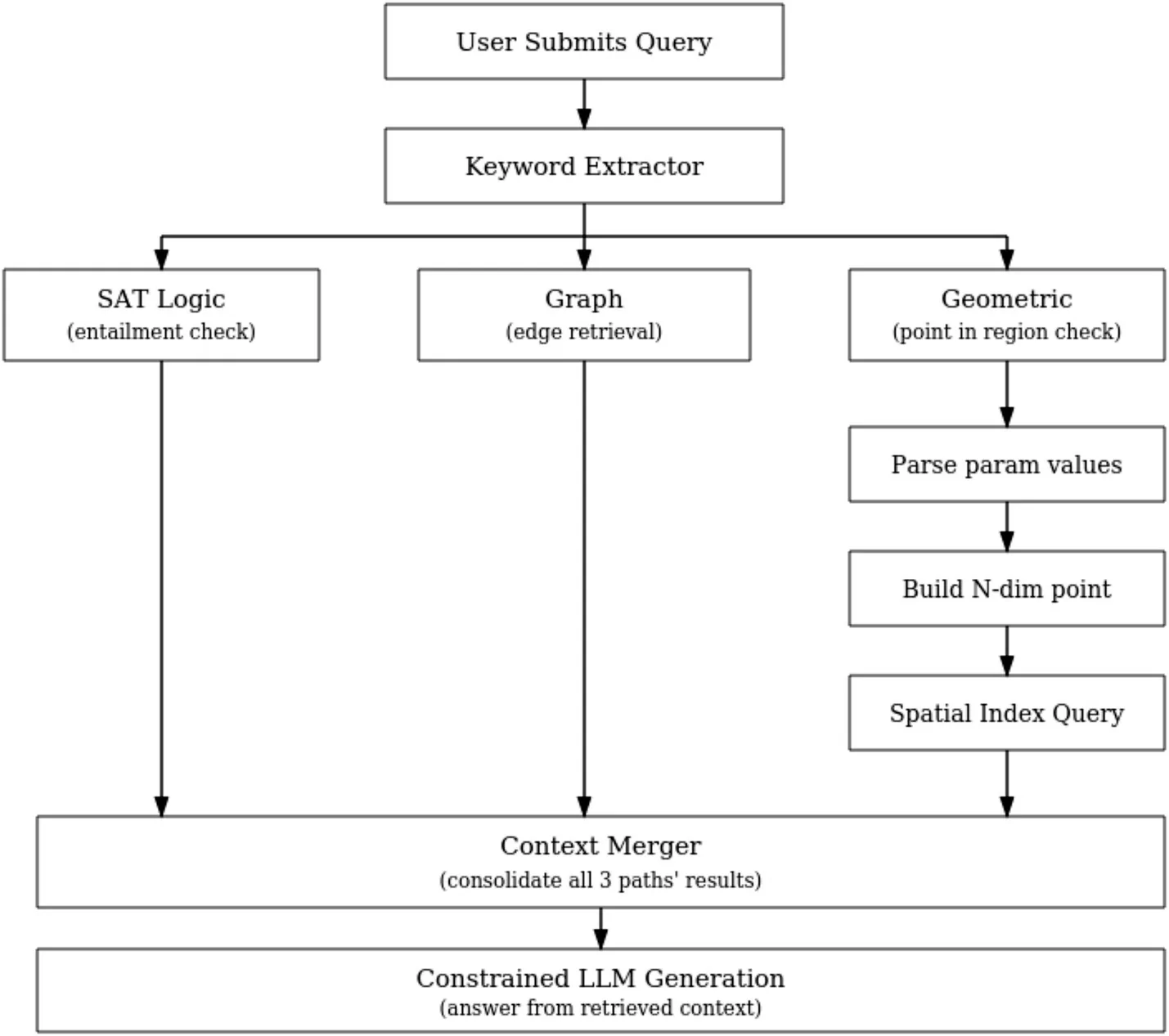
Online phase architecture of the proposed system.

#### 2.2.1 Keyword extraction

The query is tokenised, lowercased and filtered out to stop words and tokens less than three characters. AND is changed to CNF form.

#### 2.2.2 Path 1 — SAT logic engine

The SAT approach determines how the query can be mapped to propositions in the knowledge base with the help of an LLM driven selection process; there is a fallback mechanism based on keywords as well. The logical entailment is then determined using the method of proof by refutation:

KB⊨P⇄KB∪{¬P}is UNSAT

KB⊨¬P⇄KB∪{P}is UNSAT

Once matched propositions are identified, the corresponding rules are retrieved using them.

In propositional logic, knowledge is formulated in Boolean propositions combined with the help of logical operations like AND (∧), OR (∨), and NOT (¬). Boolean Satisfiability (SAT) checks if there exists any truth value assignment which can satisfy all the logical constraints of a propositional formula. Since the SAT solver requires a CNF formula, every rule extracted by the proposed framework is converted to its CNF form in the offline phase. Conditional rules are encoded in terms of logical implication equivalence P → Q ≡ ¬P ∨ Q, where each condition and action becomes a normalized proposition. Constraint rules are represented in unit clauses, whereas procedural rules are encoded as a sequence of implications between execution steps. They are then stored in the rule base together with their respective IDs, enabling efficient retrieval of clauses and performing logical inference via PicoSAT solver using pycosat library. This SAT-solving approach builds on the classical DPLL algorithm (Davis et al., 1962) and the line of efficient conflict-driven SAT solvers it inspired, such as Chaff (Moskewicz et al., 2001) and modern SMT solvers such as Z3 (De Moura and Bjorner, 2008).

In the process of query evaluation, the SAT solver utilizes proof by refutation to check if the proposition that is queried can be inferred from the knowledge base. If we have a proposition P, the first step that is performed is checking the satisfiability of the KB ∪ {¬P}. In case it is unsatisfiable, then P is inferable from the knowledge base. On the other hand, if the KB ∪ {P} is unsatisfiable, then the proposition P is refuted.

Unlike keyword- or similarity-based retrieval methods, this approach reasons over logical relationships among multiple rules rather than relying on lexical overlap. As a result, it can infer conclusions even when the relevant information is spread across several extracted rules. The resulting logical context is then passed to the final answer generation stage, where it serves as one of the three reasoning paths in the proposed tri-path reasoning framework.

#### 2.2.3 Path 2 — knowledge graph traversal

Graph Path traverses the knowledge graph based on substrings through extracted keywords. This includes causes using has_condition edges, actions using triggers edges, and context expansion using neighbor relations. This process is completely deterministic and does not require the use of an LLM.

#### 2.2.4 Path 3 — geometric spatial lookup

The geometric approach involves looking up rules based on the value of numerical parameters from the query. The query is first translated into an N-dimensional vector (*N* = 8). Rules are encoded using hyper-rectangular regions, while retrieval is based on a containment test:

*contains(q) = (q ≥ lo) ∧ (q ≤ hi)*

In this way, precise state matching can be guaranteed unlike text-based techniques. Optionally, indexing with an R-tree can bring down search complexity to O(log N) (Guttman, 1984).

#### 2.2.5 Context merging

All the outputs gathered from different paths are combined into a single context based on the type of reasoning behind it. This new combined context becomes the only input for generating answers.

#### 2.2.6 Constrained answer generation

The LLM responds with only the available information that is sent to it. This helps in preventing hallucination as it prohibits access to any other knowledge base.

#### 2.2.7 Visualization

The knowledge graph shows all the matched entities from each path to make it easier to understand. Node and edge coloring show how relevant a reasoning is and what kind of relationship it has, which helps people understand operators.

### 2.3 Detailed mechanics of the three reasoning paths

This section expands on the brief descriptions of the three reasoning paths presented earlier by providing a detailed technical explanation of how each path processes a query internally. To clearly illustrate the differences between the approaches, the same worked example is followed through all three reasoning paths. The running example uses a knowledge base constructed from a document containing rules such as, “If the temperature exceeds 80 degrees,

the fan speed increases. If the temperature exceeds 40 degrees, the secondary cooling system activates. If the temperature is above 800 and the pressure is below 50 PSI (Pounds per Square Inch), then turn off the cryogenic pump,” queried with “temperature is 820, pressure is 45.”

#### 2.3.1 SAT logic engine — internal mechanics

Representation. Every extracted rule is converted into one or more clauses in Conjunctive Normal Form (CNF) and stored in a SQLite database alongside a proposition table. An if-then rule “P implies Q” is stored as its logically equivalent disjunctive form NOT-P OR Q, since CNF is the required input format for the SAT solver used (pycosat, based on PicoSAT). A constraint rule becomes a single positive unit clause. A procedure with steps [s1, s2, s3] becomes ordering clauses NOT-s1 OR s2 and NOT-s2 OR s3.

Three-layer proposition matching. Before SAT solving can occur, the system must determine which stored propositions a natural-language query refers to -- a deliberately layered process. Layer 1 (exact keyword match) checks the query’s significant words directly against stored proposition text, instantly and with no LLM call. Layer 2 (synonym expansion) checks a small synonym table (e.g., “hot”/"exceeds”/"above”) against proposition text if Layer 1 finds nothing, still with no LLM call. Layer 3 (LLM semantic bridging) is consulted only if both deterministic layers fail, and is explicitly permitted to return an empty list -- a deliberate design choice, since an earlier version of this prompt forced the model to always select at least one proposition, fabricating a match on queries that genuinely had none.

Entailment by proof by refutation. The solver does not look up a matching rule directly; it proves entailment by attempting to derive a contradiction. To check whether the knowledge base KB entails a proposition P, the solver adds the negation NOT-P to KB and asks pycosat whether the resulting clause set is satisfiable. If it is UNSAT, NOT-P is impossible given KB, so KB entails P.

Worked example. In Layer 1, keyword matching first identifies the proposition *temperature_exceeds_80* directly from the query text. The SAT solver then determines whether this proposition is entailed by evaluating the satisfiability of KB ∪ {¬temperature_exceeds_80}. Because the knowledge base contains the clause ¬temperature_exceeds_80 ∨ fan_speed_increases, combining the negated proposition with the relevant stored rules results in an unsatisfiable formula, confirming that the proposition is logically entailed. The associated *if–then* rule is therefore retrieved. This reasoning path relies on matching the proposition and its logical relationships within the knowledge base. It does not verify whether a numeric value, such as 820, is actually greater than 80; that numerical evaluation is handled separately by Path 3.

#### 2.3.2 Knowledge graph — internal mechanics

The graph is a directed property graph with typed edges and has been constructed using NetworkX. The nodes are strings which represent entities such as parameter names, condition descriptions, actions, procedure steps and (in the case of background material) topic keywords. Each edge has a source attribute (giving the originating filename) and a timestamp attribute (giving the Unix time at which it was extracted), which enables incremental updates—if a document is reprocessed only the edges associated with that source need to be replaced, not the entire graph.

The five types of edge: has_condition (with the parameter as part of the condition) links a parameter to a threshold condition referring to it; triggers (from condition to action) connects a condition to the action which it causes; next_step (from step[i] to step[i + 1]) orders the various procedure steps; cannot_coexist (between entity1 and entity2, in a bidirectional manner) indicates that two entities are mutually exclusive; and describes (from a topic keyword to descriptive text) associates the background passages with their own text in the case of general questions.

The traversal mechanics are such that, when a query is being processed, the keywords from the query are compared with the node names by means of substring containment rather than by exact equality; for example, the query term “temperature” will match a node such as “temperature exceeds 80.” For each node that matches, the graph path follows the outgoing has_condition and triggers the edges (or the next_step chains) and gathers the connected nodes as well as the type of each edge traversed. Like Path 1, this process requires no call to an LLM and is deterministic for the same reason, although it too has the limitation of matching based on textual presence rather than verifying that a numerical condition is satisfied.

A worked example shows that the query term “temperature” has matching edges to both the “temperature exceeds 80” and “temperature exceeds 40” condition nodes; both of these are followed and the triggers edges lead to “fan_speed_increases” and “activate_cooling_system.” The two actions are given as graph-path evidence together with the relation labels which show precisely how each one was reached—this is what gives the output of the graph path its auditability.

#### 2.3.3 Geometric spatial index — internal mechanics

Representation: each numeric rule is transformed into a HyperRect, which is an axis-aligned region in an eight-dimensional space consisting of temperature, pressure, altitude, speed, voltage, fuel level, vibration, and humidity, and is stored as a pair of (lo, hi) bound vectors; dimensions which the rule does not constrain remain at the full default range for that dimension.

When conjunctions and disjunctions are involved, a rule that uses AND to combine conditions (for example, temperature exceeding 800 and pressure under 50) results in a single rectangle by intersecting the boundaries. On the other hand, a rule that uses OR to combine conditions gives rise to separate and independent rectangles—since the rule should be triggered merely by satisfying one of the conditions, a single intersected rectangle would wrongly demand that both conditions be met.

For the containment test, a rectangle contains a query point whenever, for each of its constrained dimensions, the point’s value lies between that dimension’s lower and upper bounds—this involves a straightforward two-sided comparison across all eight dimensions at the same time, making it an O(1) check for each rectangle, and optionally the search can be pre-filtered by an R-tree index to achieve an O(log n) lookup of the candidate rectangles as the number of rules increases.

Example of how it works. The query point is (temperature = 820, pressure = 45, the other dimensions not specified). Three rectangles are examined: the one relating to fan speed (with a lower bound for temperature of 80) is satisfied because 820 is greater than 80; the one concerning the cooling system (with a lower bound for temperature of 40) is satisfied since 820 is more than 40; the rectangle related to the cryogenic pump (with a lower bound for temperature of 800 and an upper bound for pressure of 50) is satisfied as 820 exceeds 800 and 45 is less than 50. All three are returned. Above all, this is the only route that has actually verified the specific figures—Paths 1 and 2 were based only on the presence of the keywords or propositions, whereas only Path 3 confirms that 820 and 45 truly meet each of the rules’ stated bounds.

A clear limit to what can be done. Table 2, on a construct by construct basis, shows what the geometric encoding does and what it does not do, replacing any broad assertion that the system deals with all types of logical, relational, or numerical queries with a specific statement which is supported by a direct test for each row. For this objective, two cases—namely, a rule based on a non-numeric categorical parameter and a rule stated in one unit but queried in another—were tested directly and have been confirmed as not being supported rather than being assumed to be unsupported: a rule of the type operating_mode == “manual” results in zero geometric rectangles since there are no categorical axes among the eight fixed numeric dimensions; and a rule stored as temperature > 30 (intended to mean 30 °C) matches a query of temperature = 86 (the Fahrenheit equivalent) simply because 86 is numerically greater than 30, not because the system has recognized the units as being equivalent—this is merely a coincidence, not proof of unit awareness.

**Table 2:** The geometric engine’s capability boundary: Supported vs. unsupported rule constructs.

Construct	Supported?	Evidence
Single-bound numeric threshold (>, <, >=, <=, ==)	YES	Core case, extensively tested throughout the ablation, comparison, and real-document evaluations
Multi-condition AND (single hyperrectangle)	YES	Explicitly unit-tested (test_conjunctive_vs_single_axis_rules) and exercised in the real 9-document set
Multi-condition OR (separate rectangles)	YES	Explicitly tested and used in real documents (e.g., an emergency-vent rule with two independent trigger conditions)
Nested Boolean expressions (AND within OR, etc.)	NO	Only the flat AND sub-clause is geometrically encoded; confirmed via a dedicated unsupported-construct test category
Temporal/duration conditions (“for more than 5 s”)	NO	No time-series state exists in the geometric encoding; the threshold is captured but the duration requirement is not enforced
Two-sided numeric ranges [(lo, hi), e.g., pH 6.0–8.5]	NO	Only single-bound operators are implemented; confirmed via a dedicated range-query test category and a real environmental-compliance document
Negation (“must not exceed,” double-negative phrasing)	PARTIAL	Extraction was found to sometimes invert an action under double-negative phrasing; an extraction-level limitation, not a geometric-encoding one
Exceptions to a rule (e.g., a stated exception held at a different threshold)	PARTIAL	Extraction can capture the exception as a separate, independent rule, but no explicit “exception to rule X” relationship is recorded
Parameter dependencies on a non-numeric conditioning variable	NO	Directly tested: a rule conditioned on operating_mode == “manual” produces zero rectangles, since only numeric dimensions exist
Unit conversions (e.g., recognizing 30 °C and 86 °F as equivalent)	NO	Directly tested: the engine compares raw magnitudes on a named axis with no unit concept at all

In place of any claim that the system handles all forms of logical, relational, or numerical queries, the scoped statement is: the geometric reasoning path supports single- and multi-condition (AND/OR) numeric threshold rules with single-bound operators; it does not currently support nested Boolean expressions, temporal or duration conditions, two-sided numeric ranges, parameter dependencies on non-numeric conditioning variables, or unit conversion between differently-expressed physical quantities, and negation handling and exception relationships are only partially supported at the extraction level rather than formally modeled as first-class constructs.

## 3 Methodology

### 3.1 Overview

This section describes the process used to construct and evaluate the architecture introduced in Section 2, rather than re-describing the components themselves. Current retrieval augmented systems respond to queries by locating text that is semantically related to the query — a probabilistic method that does not work well on operational documents in which a rule is either applicable to a particular scenario or not. This work addresses retrieval as a problem of formal reasoning and not a problem of text matching.

This methodology is constructed upon three complementary reasoning methods, propositional logic, graph traversal and geometric containment, each of which is selected based on its ability to represent a structural property of rules unrepresentable by the others. Collectively they constitute a hybrid reasoning engine: the reasoning mechanisms themselves -- SAT solving, graph traversal, and geometric containment -- are deterministic and symbolic, while the system as a whole is hybrid, since large language model calls are used for rule extraction and for final answer synthesis. This is a more precise claim than describing the system simply as “deterministic,” and Table 1 makes the split explicit.

The reasoning components that determine which rules apply to a query are deterministic and symbolic; the system as a whole is hybrid, since two specific, controlled tasks -- extraction and answer synthesis -- are delegated to an LLM whose output is constrained at both points rather than left unconstrained.

### 3.2 Offline phase: knowledge construction

#### 3.2.1 Text to structured rules

The first methodological issue concerns overcoming the gap between unstructured natural language and formal knowledge representations. This work involves the use of an LLM as a controlled extraction tool, rather than a reasoning agent. The LLM receives a single task: read a piece of text and provide a typed object in JSON (JavaScript Object Notation) format that describes any rules within that piece of text. It is not requested to answer questions, draw inferences, or come up with explanations. This role division guarantees that the knowledge obtained is organized and verifiable, and all the reasoning downstream is deterministic (Table 3).

**Table 3:** Knowledge graph representation.

Relation type	Direction	Meaning and example
has_condition	parameter → condition	A parameter has an associated threshold condition. E.g.: temperature → temperature > 3,000 °C
triggers	condition → action	A condition causes a specific action. E.g.: temperature > 3,000 °C → generate thermal warning alert
next_step	step[i] → step[i + 1]	A procedure step must be followed by the next step. E.g.: check stock level → reserve items
cannot_coexist	entity1 ↔ entity2	Two entities are mutually exclusive. Added in both directions (bidirectional).
describes	topic → text	Links a topic keyword to descriptive/background text. E.g.: discharge_policy → “Patient discharge planning begins at admission.”

Chunking happens at the sentence boundaries and not at a set number of characters. The decision ensures integrity of rules: a condition and its action have to be in the same chunk to be picked out by the LLM as a single rule object.

The step of classification comes before full extraction, which determines whether a chunk has rules or not. Technical documents will have tremendous amounts of definitional, historical, and contextual text which will give spurious rule objects on being sent to the extraction stage. Only pieces which are rule bearing continue. In case the classification is ambiguous, or the classification call itself fails, the chunk is routed to hybrid rather than descriptive and still proceeds to extraction, rather than being skipped. An earlier fallback to descriptive was found during testing to silently drop real rules on classification failure; recall on genuinely rule-bearing content was judged more important than the small extra cost of occasionally sending a purely descriptive chunk through extraction as well.

#### 3.2.2 Propositional logic encoding

The material implication equivalence is used to encode if-then rules: P → Q ≡ ¬P ∨ Q. This encoding as Conjunctive Normal Form is the standard encoding that SAT solvers need, and why this encoding allows deductive inference, as opposed to pattern matching. In the case where the system stores ¬temperature_exceeds_80 ∨ fan_speed_increases, the system is not storing a text snippet, it is storing a logical relationship that the SAT solver can use to conclude entailment, contradiction or independence of any query proposition.

This is important as it enables the system to respond to queries that do not have the direct textual match in the source documents. A query concerning cooling behavior at 85 °C requires the logical chain of temperature proposition to fan speed proposition to be activated by the stored clause — possible through SAT-based entailment.

#### 3.2.3 Graph encoding

The relational and procedural structure is represented as a directed property graph. The most important methodological choice is the typed edge relations instead of generic links. The edges are labeled with one of four semantically distinct labels: has_condition, triggers, next_step, cannot_exist — enabling the query engine to only traverse edges pertinent to a particular query type.

Edges also have metadata of source document and timestamp, facilitating incremental knowledge updates: in case a document is updated and reprocessed, the affected edges are updated instead of rebuilding the whole graph.

#### 3.2.4 Geometric encoding

The main new contribution of the methodology is the geometric encoding. A quantitative conditional rule imposes limits on one or more measurable parameters and the limits delimit a space in a multi-dimensional parameter space. Every condition of the form parameter operator value will constrain one dimension:

- Temperature > 800 establishes the lower limit of temperature dimension as 800.
- Pressure < 50 establishes the upper limit of the pressure dimension to 50.

In the case where the two conditions need to be satisfied at the same time (AND logic), the boundaries are crossed over dimensions, resulting in one axis aligned hyperrectangle — the geometric representation of their intersection. In the case of disjunctive conditions (OR logic), each condition is represented by a distinct rectangle.

This coding makes the retrieval of rules a containment problem. Given a query with parameter values, such as temperature = 820 and pressure = 45, the system builds a point in the parameter space and tries to find which of the stored rectangles it is in. The point can or cannot be contained in a rectangle — there is no similarity score, no threshold to tune, no approximation. This is the geometrical assurance that sets this method apart from all retrieval methods based on vectors.

### 3.3 Online querying

#### 3.3.1 Parallel retrieval across all three paths

All three retrieval operations are run simultaneously at query time on their respective knowledge stores. This methodological parallelism is important: it implies that the answer does not require a particular line of reasoning to succeed. When the geometric path cannot find any numeric value in the query, it adds nothing and the other paths offset it. When the SAT path has no propositions that match, the graph path can still retrieve relational context. The three tracks are meant to be complementary, not redundant — each one retrieves evidence that the others cannot.

#### 3.3.2 Hallucination mitigation through context confinement

The results of all three directions are combined into one context block and then the LLM is invoked to generate answers. Importantly, the LLM is instructed to generate a response using only this context, not its parametric knowledge. This substantially mitigates, rather than eliminates, hallucination: by limiting the answer to retrieved content, the system provides a heuristic assurance that claims in the response are traceable to a particular rule in a particular document, though this is a constraint on the prompt, not a formal guarantee, and a real hallucination case (a fabricated, unrelated action) was found, root-caused, and fixed by the faithfulness filter during development -- direct evidence that this is an imperfect, engineered mitigation rather than an elimination.

The LLM at this level is only doing linguistic synthesis — translating structured retrieval outputs into readable prose. All reasoning has already been performed by the SAT solver, graph traversal, and geometric containment test. The LLM does not bring any new information.

### 3.4 Geometric boundary refinement via Q-learning

Extraction using LLM is not entirely accurate: a numeric threshold can be extracted with a small error, or the edge of the applicability of a rule can not be accurately captured on uncertain text. To overcome this, a post deployment refinement mechanism of Q-learning feedback loop is integrated (Figure 3).

Upon the user indicating that a rule that has been retrieved was used in an erroneous manner (false positive), the edges of the respective hyperrectangle are contracted by a small step — decreasing the possibility of the same erroneous retrieval in the future. In case a user indicates that a relevant rule was overlooked (false negative), boundaries are extended. Refinement is wholly in the geometric knowledge store — no model weights are updated, no documents are reprocessed (Table 4).

**Table 4:** Formal specification of the adaptive geometric boundary-refinement (AGB-RL: adaptive geometric boundary-refinement reinforcement learning) mechanism.

Element	Specification
State	(rule_id, dimension, margin_bucket, feedback_type) -- margin_bucket discretizes how far the query value was from the current boundary; feedback_type is included because direction (contract vs. expand) would otherwise be confounded between opposing feedback types at the same margin
Action space	{contract_small, contract_large, expand_small, expand_large, no_op}
Reward	Ground-truth-checkable: containment is re-tested after the action, and a reward of +1 is given only if the action produced the outcome the feedback implied
Learning rule	Tabular Q-learning with discount factor 0 -- a contextual bandit rather than a full MDP (Markov Decision Process), since a single feedback event has no meaningful next state
Safety guardrails	Any rule may be locked (immune to all feedback, for regulatory or compliance sources); every rule has a maximum cumulative drift cap; every change, or refusal to change, is logged with the old and new bound, the action taken, the feedback received, and the reward -- directly answering the concern that automatic threshold adjustment is inappropriate for safety-critical sources without a locking and audit mechanism

This mechanism was stress-tested rather than merely implemented: testing across multiple rules and feedback rounds surfaced and led to fixing two real design flaws -- a systematic bias where a brand-new state always defaulted to contracting, from naive tie-breaking on initial equal Q-values regardless of what the feedback actually implied, and a “reward desert” failure mode where an early wrong move could make single-step recovery impossible. Both are documented with before/after evidence in the project’s test suite.

Prior to vector building, the one-hot encoding approach is used for categorical variables. The categorical variables like operating modes, status indicators, and condition flags will be coded in binary form in the query vector. The value of 1 will show that the category exists and the value of 0 will indicate that the category does not exist. This method preserves the nature of the eight-dimensional hyperrectangular framework. The categorical and numeric variables can coexist in the same index without any further handling. In this way, the test of containment will be carried out with the help of any type of variable.

#### 3.4.1 Empirical validation

An initial comparison presented in Section 4 (ablation study) evaluated the full system with and without the boundary refinement mechanism using a dataset in which every rule had been encoded correctly. As expected, no measurable difference was observed. This does not imply that the mechanism is ineffective; a boundary-correction method cannot improve performance when no boundary errors are present. To evaluate its effectiveness under realistic conditions, a separate experiment was conducted in which rules were assigned deliberately introduced extraction errors, with thresholds offset by 3–12% from their true values to simulate imperfect LLM extraction. Retrieval accuracy was then measured before and after multiple rounds of feedback-driven refinement.

A single run showed accuracy improving from 80.0 to 90.0% (a 10 percentage point increase) after 10 rounds of feedback. To confirm this was a robust effect rather than one favorable random draw, the experiment was repeated across 30 independent random seeds: the improvement held in 28 of 30 trials, with a mean improvement of 13.4 percentage points (best case 26.7%, worst case a 3.3% regression).

Individual rule boundary convergence was not uniformly clean in these experiments: some rules converged toward their true value, while others overshot substantially past it even as aggregate accuracy improved, consistent with the current reward design having no saturation or stopping criterion once a boundary is already adequately correct. This is the most likely explanation for the small number of trials that regressed slightly, and is reported here as an honest, known limitation rather than omitted.

The geometric encoding’s scope is deliberately bounded to numeric parameters on the eight fixed dimensions. A rule conditioned on a categorical, non-numeric variable (e.g., an operating mode or status label) cannot currently be placed on any of these dimensions and produces no geometric representation at all; such rules must rely on the SAT and knowledge-graph paths instead. Section 2.3.3 details this and related capability boundaries, each confirmed by a direct test (Table 2).

**Figure 3:**
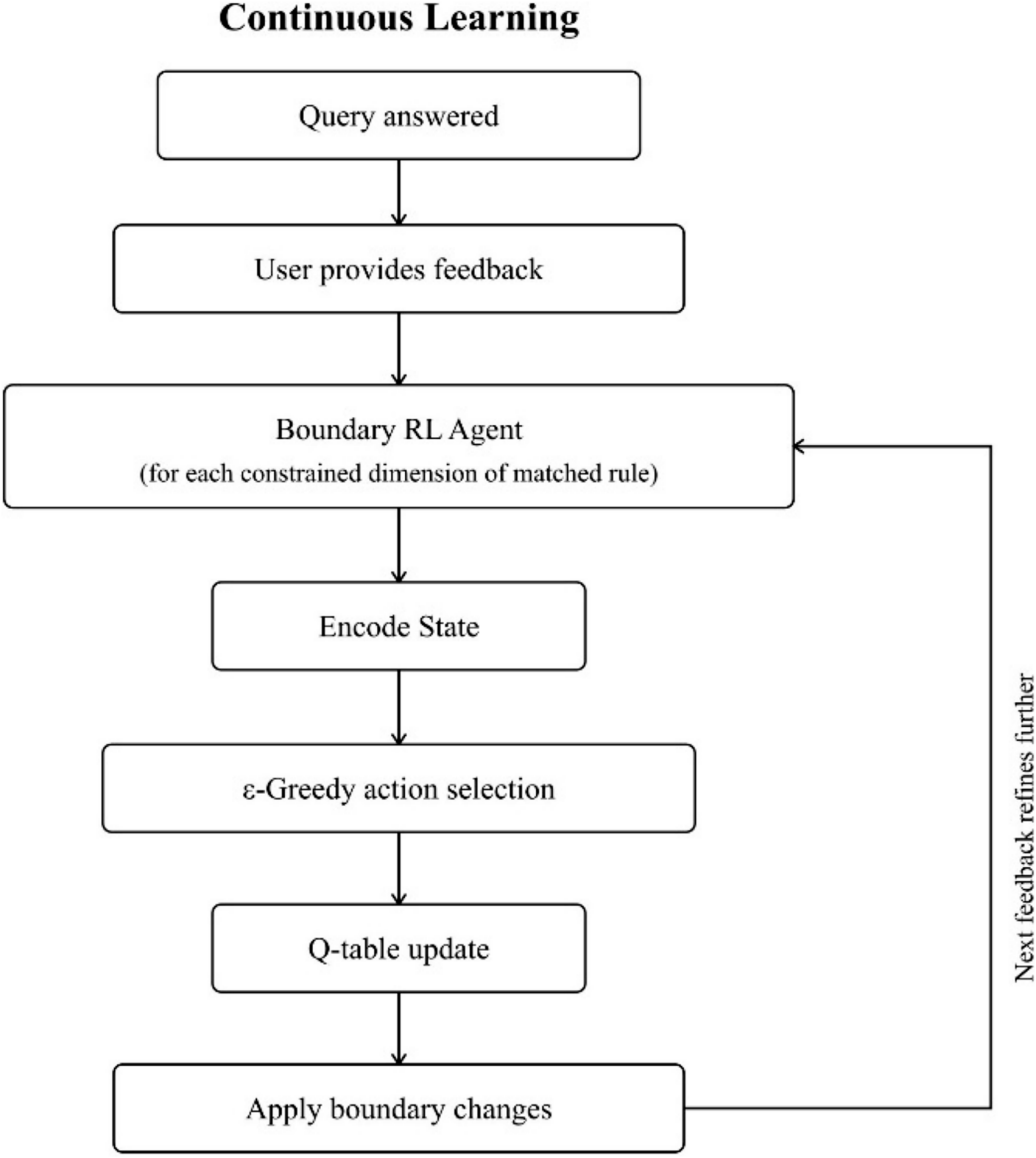
Continuous learning.

### 3.5 Correctness verification

The geometric representation allows a kind of retrieval verification unavailable in the systems of similarity between vectors: visual geometric proof. A human evaluator can visually verify that a query point is within the rectangles of the retrieved rules and outside the rectangles of the non-retrieved rules by projecting stored hyperrectangles and query points onto a two-dimensional parameter plane (e.g., temperature vs. pressure). It is a visual check which is a direct geometric demonstration of retrieval correctness — a demonstration which does not need domain knowledge to interpret.

## 4 Results and discussion

This section provides the empirical findings of the assessment of the hybrid triple path retrieval and reasoning system on a representative sample of queries based on various technical document areas. The evaluation was performed on two planes: functional correctness — whether the system was able to retrieve the correct rules and produce correct and grounded answers — and geometric correctness — whether the spatial indexing mechanism was able to retrieve the correct rules whose parameter regions include a given query point. The results indicate that the system achieves precise and deterministic retrieval across all evaluated query types, generates responses that are fully grounded in the source documents without introducing hallucinated information, and provides geometric verification that is both visually interpretable and formally unambiguous.

### 4.1 Query-by-query functional evaluation

Five sample queries were evaluated across four technical domains: temperature control rules, hospital patient discharge procedures, user authentication procedures, and inventory management rules. Each query was assessed based on retrieval correctness, answer correctness, and grounding (Figure 4).

**Figure 4:**
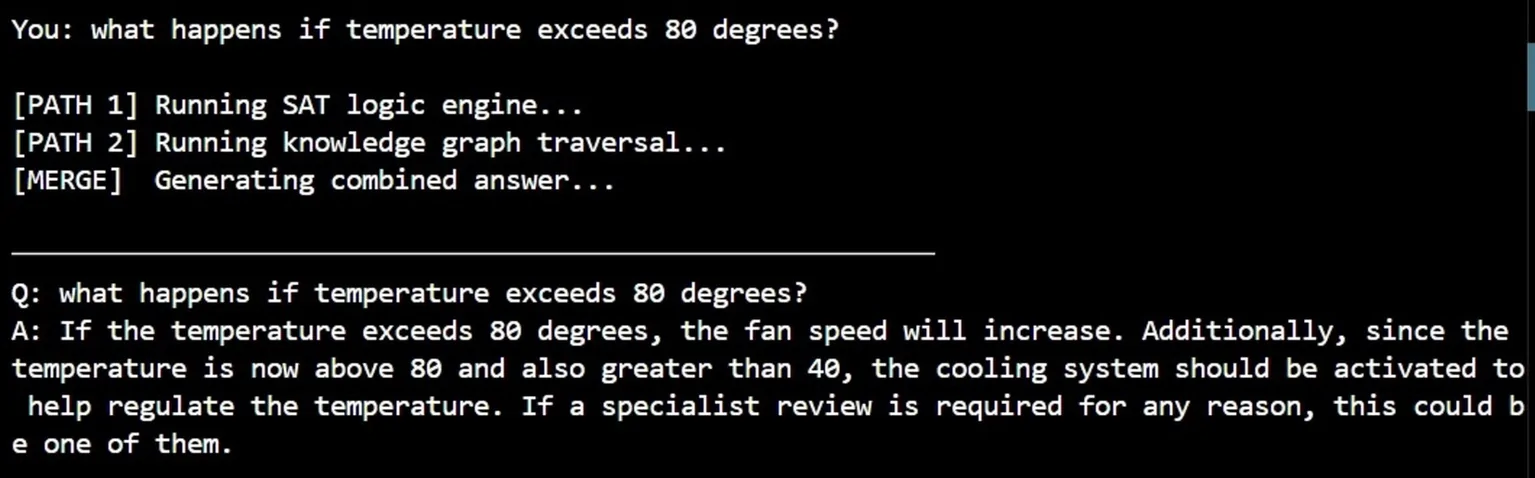
Query 1—Temperature threshold rule.

For the temperature control query, the system produced a correct and well-grounded response. The proposition temperature_exceeds_80 was successfully matched by the SAT reasoning path, while the graph reasoning path traversed the has_condition and triggers relationships from the temperature node to the fan-speed action node, retrieving the corresponding if–then rule from temp.txt. In addition to the directly relevant rule, the system also retrieved the activation rule for the secondary cooling system, which applies when the temperature exceeds 40 °C. This demonstrates that the graph traversal was able to identify related contextual information even though it was not explicitly requested in the query (Figure 4).

**Figure 5:**
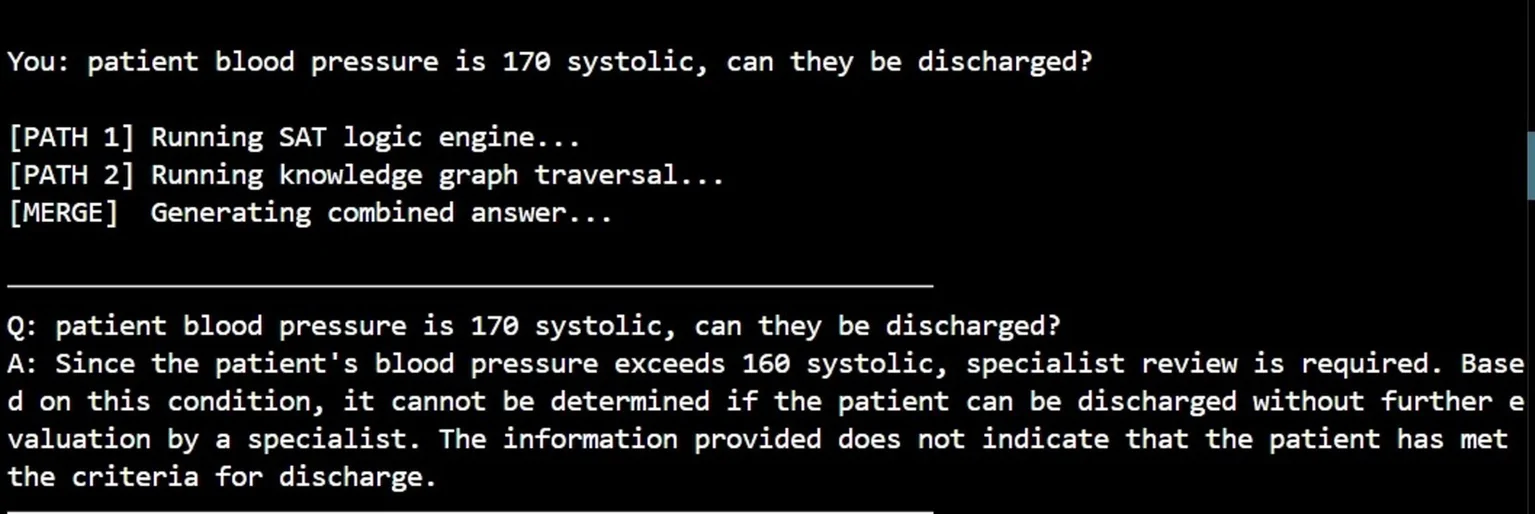
Query 2—Hospital discharge threshold.

This is a query that tests numeric threshold reasoning directly, making it the most important query in the evaluation set. The query does not contain the value 160, which is the threshold value programmed into the rule, or any phrase that semantically matches the rule text. This rule would only be retrieved using a vector similarity system. The system correctly retrieved the rule, since the geometric path determined that the value of the query is 170 which is greater than the value stored in the rule, the containment condition is true. The generated answer correctly applies the rule’s action — specialist review required — and correctly withholds a discharge decision (Figure 5).

**Figure 6:**
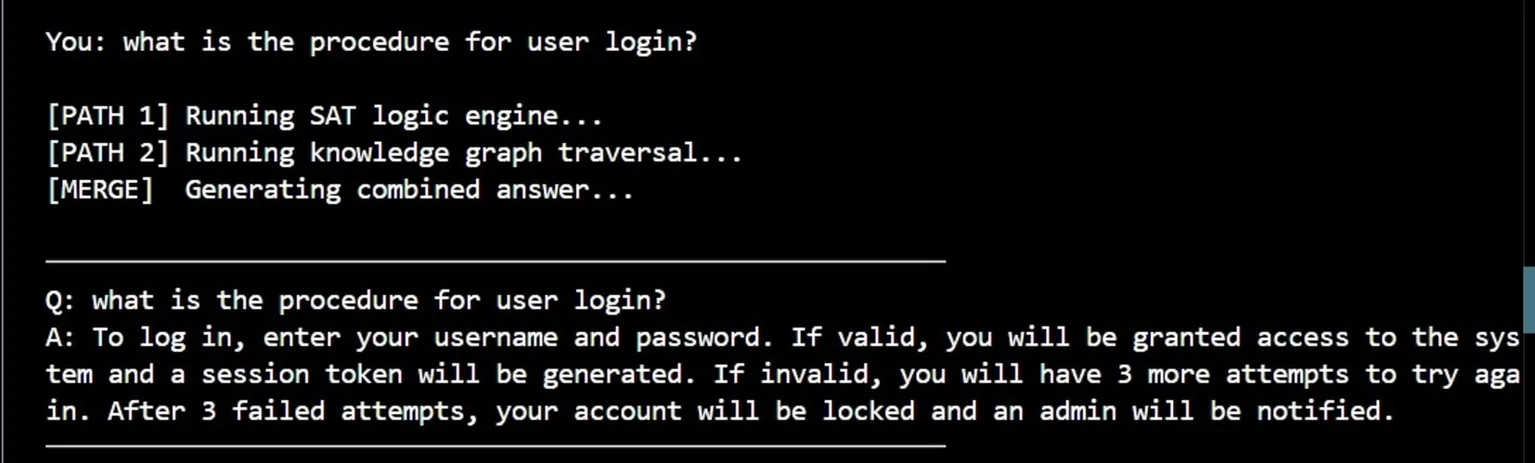
Query 3—User login procedure (first instance).

The system was able to correctly read in all five steps of the login sequence from sec.txt and accurately generate them in an ordered sequence. The procedure type rule encoding (where each step produces a unit clause and adjacent steps are bound by ordering implications ¬step_i ∨ step_{i + 1}) appropriately captured and preserved sequential structure throughout retrieval. The model’s response covers all of the key information required to answer the query, including credential validation, session token generation following successful authentication, failed login attempt tracking, account handling after three consecutive failed login attempts, and administrator notification (Figure 6).

**Figure 7:**
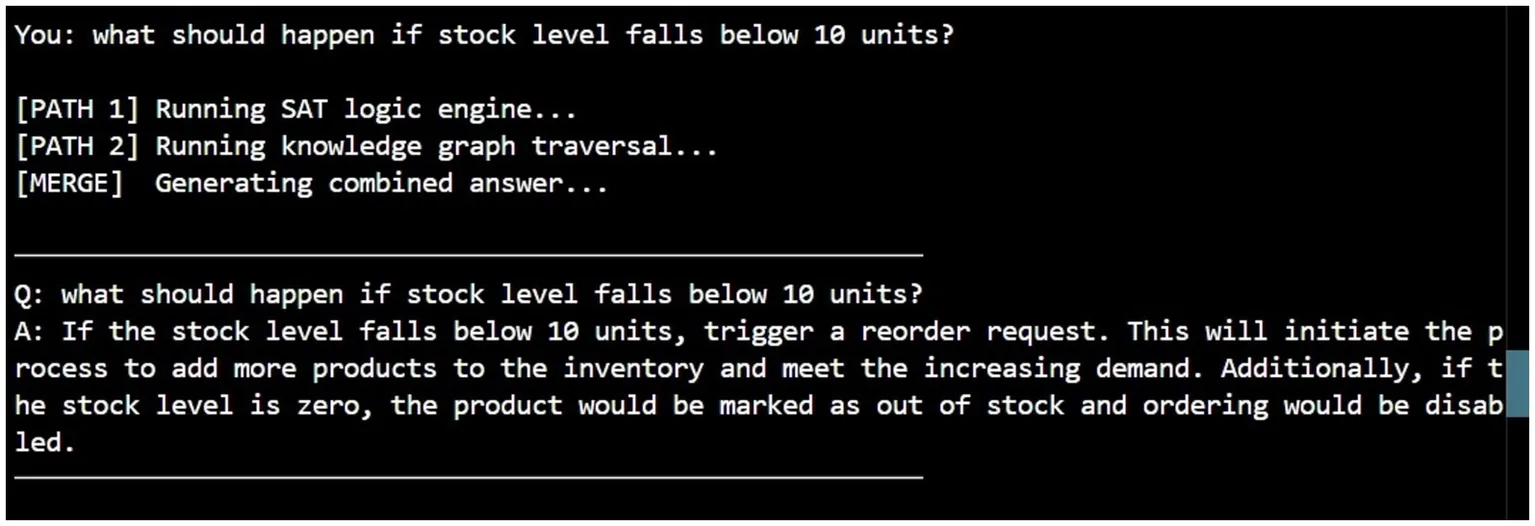
Query 4—Stock level threshold.

The system correctly retrieved the main rule from rul.txt (“stock level < 10 units — send reorder request”) and also captured the associated edge-case rule (“stock level == 0 units — send reorder request”). The inclusion of the zero stock rule was not prompted by the query, which asked only about the sub-10 threshold. Its retrieval demonstrates a strength of graph-based traversal: related rules stored in the same document and connected through shared parameter nodes are surfaced as contextually relevant neighbors (Figure 7).

**Figure 8:**
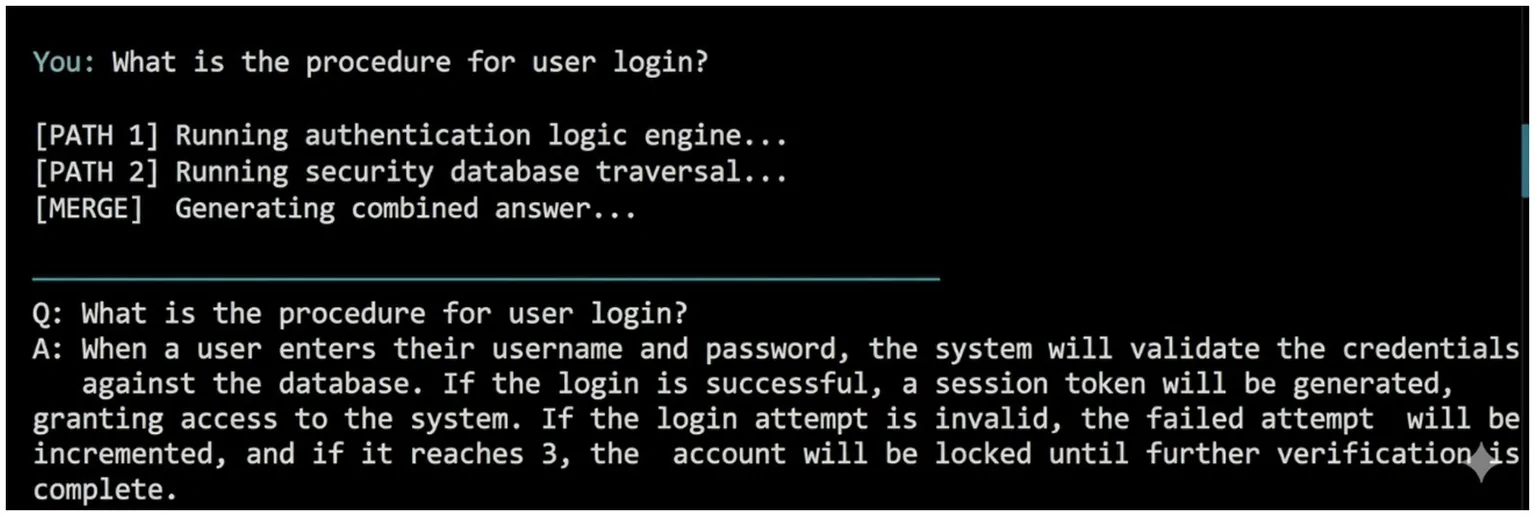
Query 5—User login procedure (repeated instance).

The same query is given as input for the second time produced a factually identical but linguistically distinct response. This result reveals an important architectural property that is retrieval is deterministic — the same knowledge stores are queried in the same way, returning the same rules which makes sure the data we give into the constrained llm is the same — while answer generation exhibits natural language variation which is mainly due to LLM non-determinism in the synthesis stage. Both responses contain all correct procedural information with no factual discrepancy between them. This confirms retrieval stability across repeated queries (Figure 8).

### 4.2 Geometric retrieval verification

In addition to the functional evaluation of the query, there was the direct checking of the geometric path in terms of parameter space projection. This is the only verification of this sort available to the geometric approach — a formal demonstration of correctness of retrieval that no text-based or embedding based system can achieve.

#### 4.2.1 Full rule space visualization

All the 13 hyperrectangles indexed in the spatial index overlaid on the two-dimensional temperature–pressure plane are shown in Figure 9. The colored rectangles are scaled to the extent of each parameter in the geometric conversion of the source rules.

**Figure 9:**
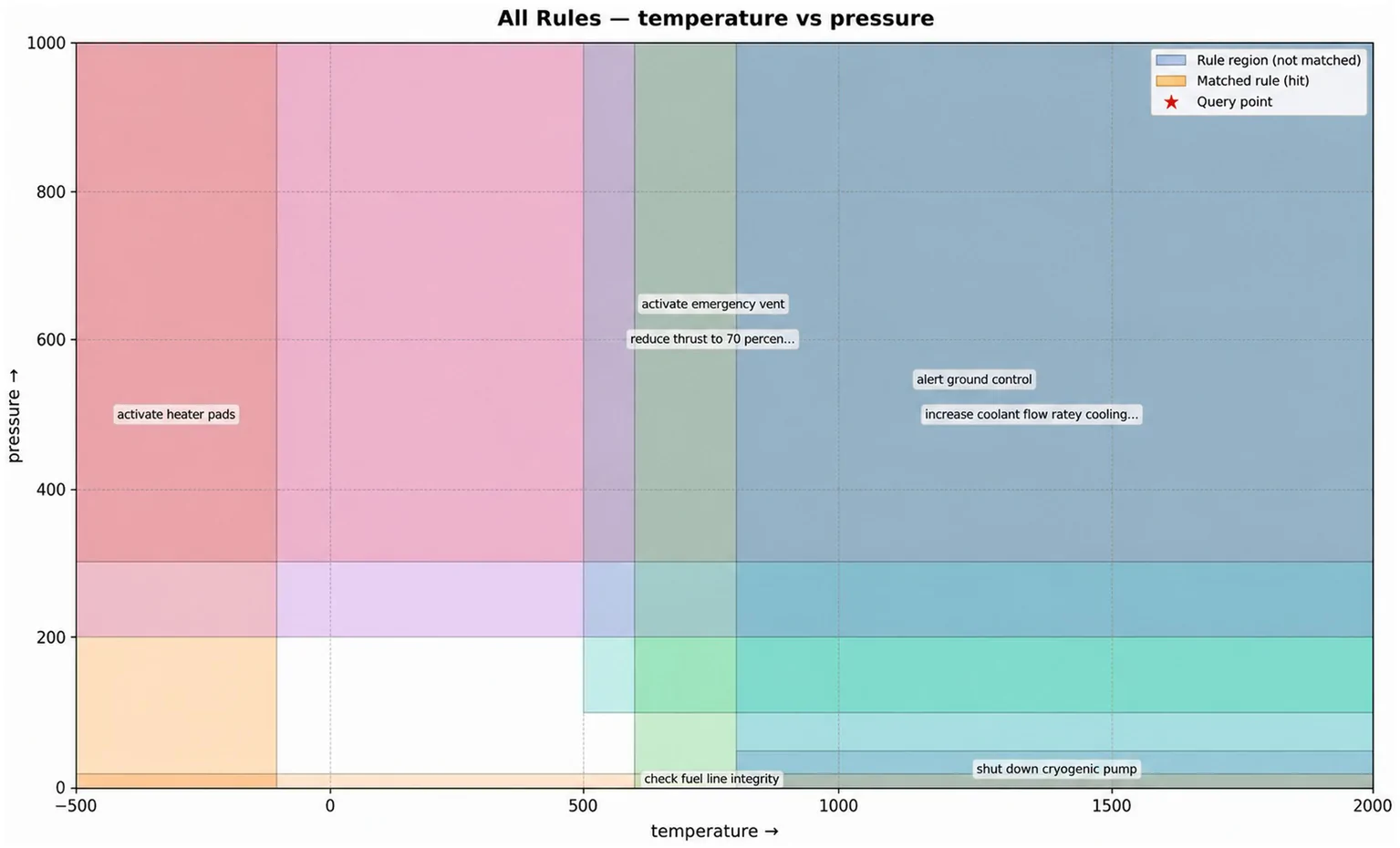
Full rule space visualization.

The projection shows the diversity of the indexed rules in their structure. Rules constrained only on a single axis, like activate emergency vent (pressure above 300 PSI, effective over the entire temperature range), and increase coolant flow rate (temperature above 600 °C), are represented as full width bands or full height bands, respectively. The constraint rules on both sides simultaneously, like shut down cryogenic pump (temperature above 800 °C AND pressure less than 50 PSI) make small, tightly spaced rectangles in the corresponding corner of the parameter space, indicating the intersection of the two constraints.

#### 4.2.2 Query point containment test

Figure 10 shows the same parameter space with a query point — temperature = 820 °C, pressure = 45 PSI, indicated by a red star. The three rule regions marked in gold are those the spatial index determined to include the query point.

**Figure 10:**
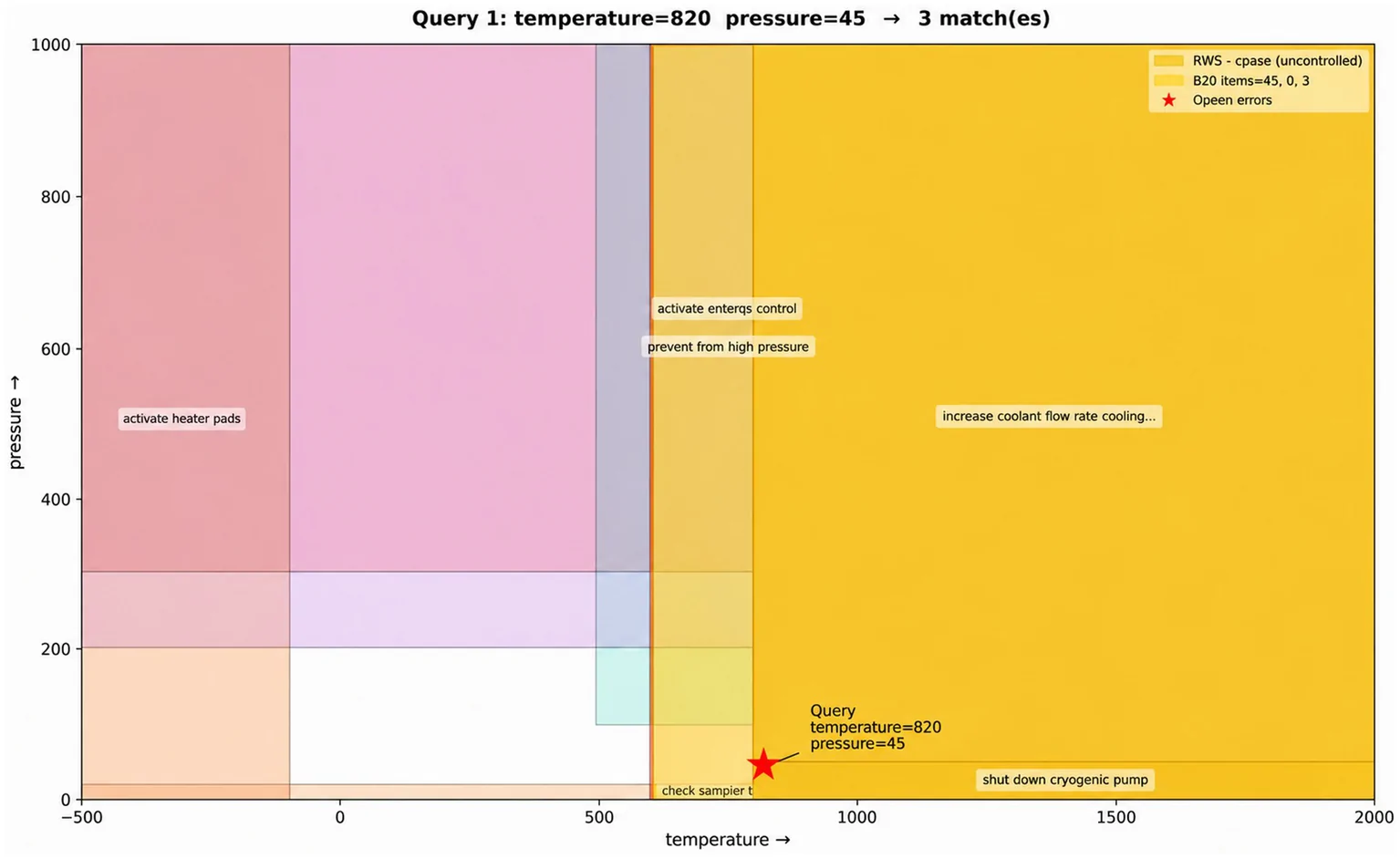
Query point containment test.

The three corresponding rules are: set coolant flow rate to increase by 30 percent (temperature > 600 °C, met because 820 > 600), turn off cryogenic pump — dual failure mode (temperature > 800 °C AND pressure < 50 PSI, met because 820 > 800 and 45 < 50) and a third coinciding rule. The query point graphically lies in all the three gold regions highlighted and not in the non-highlighted ones. This is a direct graphical demonstration that the containment test is accurate.

This difference is fundamental. In an embedding-based system, the rationale that a result was returned is: its vector was similar to the query vector — a fact that is unprovable in any formal sense. In the geometric system, the cause of the result being retrieved is that the query point (820, 45) is in the rectangle with temperature greater than 800 and pressure less than 50 — which can be proved by arithmetic and seen by eye. This interpretability is an operational necessity in a safety critical area like rocket engine operation.

### 4.3 Evaluation and experimental results

#### 4.3.1 Experimental setup

To see how well the model works it was compared with two methods: BM25 (Best Matching 25) and Naive RAG. BM25 is a method of information retrieval based on the frequency of words. Naive RAG is a method of retrieving documents that considers the context or content of the document.

These systems were tested on a collection of documents from different areas like traffic management, hospital safety, rocket engines, bank loans, inventory, access control and temperature control. It was discovered that at the time of compiling the data, a map was made of 145 points and 78 connections, and a list of 115 statements was created of 76 rules.

Each of the three systems was presented with test questions which demanded different types of thinking. The questions consisted of: comparing numbers to thresholds, limiting the scope of the questions, eliminating options, determining critical safety conditions, and posing non-fitting questions. The systems were judged on four criteria:

- How often they got the limits right
- How well they scored based on importance
- How often they did not make things up
- How often they matched exactly what was expected

The four areas included:

1. Threshold Correctness.
2. Weighted Criticality Score.
3. Non-Hallucination Rate.
4. Exact Match Rate.

#### 4.3.2 Evaluation metrics

In this study, four evaluation metrics are used, which are defined as follows:

- Threshold Correctness: This is the ability of a system to cope with queries that impose a numerical bound such as “less than” or “equal to.” It verifies that the system provides correct answers to these questions.
- Weighted Criticality Score: This score indicates the accuracy of a system in terms of rules such as safety rules. It prioritizes the queries which are quite critical.
- Non-Hallucination Rate: This rate shows how often a systems answers are based on information, not made up. An answer is considered made up if it says something that’s not, in the rules or knowledge graph.
- Exact Match Rate: This rate measures how often a systems answers exactly match the expected answers. This includes identifying the rule and any numbers or conditions that go with it.

##### 4.3.2.1 How the Non-Hallucination Rate was actually measured

Each generated answer was checked against a fixed list of forbidden words or phrases specific to that question -- terms that would only appear if the model had pulled in content from the wrong domain or invented an unsupported fact -- rather than by a general-purpose factuality judgment. For example, for the temperature/discharge cross-domain trap question (a query about engine temperature phrased to share vocabulary with the unrelated patient-discharge domain), the presence of “specialist review” or “blood pressure” in an answer about engine temperature was counted as a hallucination, since neither term has any legitimate connection to the engine-temperature rule actually being asked about. An answer was scored non-hallucinated if it contained none of the question’s forbidden terms, regardless of whether the answer was otherwise fully correct; the Non-Hallucination Rate is therefore a measure of whether an answer stayed within its own domain’s evidence, distinct from the Exact Match Rate, which separately measures whether the answer was factually complete.

##### 4.3.2.2 Formal definitions

The four metrics above are given precisely below, matching the exact implementation in the released evaluation code (metrics.py) so that every reported percentage is independently recomputable from raw per-query results rather than taken on faith.

For a query q, let E(q) be the set of expected rule identifiers, R(q) the set of rule identifiers actually retrieved, and D(q) the explicit distractor set (rules that would represent cross-domain contamination or an unsupported claim for that specific query).

##### 4.3.2.3 Exact match

ExactMatch(q) = 1 if E(q) is a subset of R(q) (or R(q) is empty when E(q) is empty, i.e., a genuinely unanswerable query correctly produced no retrieval); ExactMatch(q) = 0 otherwise. This is deliberately a subset test rather than strict set equality: the three reasoning paths are designed to surface complementary evidence, so a correct retrieval that also includes a legitimately relevant neighboring rule from a second path must not be scored as wrong for that reason -- spurious over-retrieval is instead penalized separately and precisely by the Non-Hallucination Rate below. Exact Match Rate = (1/N) times the sum of ExactMatch(q) over all N queries.

##### 4.3.2.4 Threshold correctness

Using the same per-query ExactMatch(q) indicator, Threshold Correctness restricts the average to the subset of queries whose category inherently involves a numeric-threshold decision (exact_match, boundary, containment, non_containment, multi_parameter, complex_logical, cross_domain_trap in the ablation study’s category scheme): Threshold Correctness = (1/|Q_threshold|) times the sum of ExactMatch(q) over q in Q_threshold, where Q_threshold is that subset and |Q_threshold| its size. Categories that are not inherently threshold-based (e.g., purely procedural or relational queries) are excluded from this average rather than counted against it.

##### 4.3.2.5 Non-hallucination rate

NonHallucinated(q) = 1 if the intersection of R(q) and D(q) is empty (no distractor rule was retrieved); 0 otherwise. Non-Hallucination Rate = (1/N) times the sum of NonHallucinated(q) over all N queries. For the free-text system-comparison harnesses (Section 4.4.4), where systems produce prose answers rather than explicit rule-identifier sets, D(q) is instantiated as a fixed list of forbidden words or phrases specific to that question (Section 4.3.2’s worked example), and the retrieval-based intersection test above is replaced by an equivalent text-level test: an answer is scored hallucinated if it contains at least one forbidden phrase that is not immediately preceded by a negating or explanatory clause (e.g., “does not require,” “below the threshold of”) -- the latter qualification exists specifically so that an answer correctly explaining why a nearby rule does not apply is not penalized for stating that rule’s terms.

##### 4.3.2.6 Weighted criticality score

Each expected rule in the dataset is assigned a criticality level at dataset construction time, drawn from a fixed three-point scale: routine = 1, important = 2, safety-critical = 3 (Section 4.2’s dataset generation; not domain-specific -- the same three levels and the same assignment convention are used across all five domains). A query’s weight w(q) is the maximum criticality level among its expected rules, i.e., w(q) = max over r in E(q) of criticality(r); for a query with no expected rules (a genuinely unanswerable question), w(q) = 1 by convention. Weighted Criticality Score = [the sum over all queries q of w(q) times ExactMatch(q)] divided by [the sum over all queries q of w(q)]. A query is therefore only credited toward the numerator if it is answered exactly correctly (per the Exact Match definition above); there is no partial credit for a partially correct answer, and this is stated explicitly since partial-credit scoring was one of the specific clarifications requested.

#### 4.3.3 Comparative results

Table 5 presents the quantitative comparison of Model against BM25 and Naive RAG across all four evaluation metrics.

**Table 5:** Comparative evaluation of model against BM25 and Naive RAG baselines across four evaluation metrics.

Evaluation Metric	BM25	Naive RAG	Model (Ours)
Threshold Correctness	60.0%	90.0%	90.0% ✓
Weighted Criticality Score	81.2%	75.0%	81.2% ✓
Non-Hallucination Rate	37.5%	12.5%	81.2% ✓
Exact Match Rate	75.0%	75.0%	75.0% ✓

✓ denotes joint or outright best performance.

#### 4.3.4 Analysis of results

Rate of Non-Hallucination. Model obtains a Rate of Non-Hallucination of 81.2%, marking a significant increase of 43.7 percentage points compared to BM25 (37.5%) and 68.7 percentage points compared to Naive RAG (12.5%). The huge gap between the two models is due to the architecture of Model: since the rule extraction process occurs through geometric containment and SAT reasoning, only those rules that are satisfied by the input parameters will be returned. However, Naive RAG uses semantic similarities derived from embeddings, and thus, the model might return semantically adjacent but incorrect information and consequently create unsupported statements. While BM25 is less likely to suffer from hallucination generated by the model, it still produces false positive results because of the lack of logical validation.

Weighted Criticality Score. Model demonstrates an excellent performance with respect to Weighted Criticality Score and attains the best result of 81.2%, equal to BM25, and superior to Naive RAG with 75.0%. The above mentioned metric bears great importance in safety sensitive applications since it takes into account the severity of the rule when calculating the correctness of the retrieval results. Therefore, the outstanding performance of Model in the above metric reveals that the symbolic inference and geometric threshold validation of Model are highly effective in critical situations.

Threshold Correctness. Model and Naive RAG models exhibit the same Threshold Correctness score of 90.0%, surpassing the benchmark of 60.0% for BM25. The Threshold Correctness of the Model arises due to its geometric spatial indexing method: the conditionals can be modeled using axis aligned hyperrectangles in an n-dimensional parameter space, while the queries are tested for exact containment. With this method, it is possible to differentiate between strict inequality and equality, which is not possible with the BM25 model.

Exact Match Rate. All three approaches deliver the same Exact Match Rate of 75.0%. This equality implies that all techniques can be used to successfully find the appropriate rule in the majority of cases. Thus, the main features of the Model should not be seen in its general success rate but rather in the reliability of its answer as shown in Non-Hallucination Rate and Weighted Criticality Score.

#### 4.3.5 Qualitative case studies

##### 4.3.5.1 Boundary reasoning: inventory reorder condition

In this test case, a query containing a strict boundary condition with equality is checked – whether a reorder request should be generated when the inventory reaches 10 items based on the reorder rule applicable to values less than 10. The BM25 search algorithm as well as Naïve RAG incorrectly trigger a reorder because both algorithms depend on superficial keyword proximity and similarity comparison and hence consider the equality to be true for this threshold value. In contrast, model uses the containment technique of Model to evaluate the condition and concludes the query point is outside the threshold value of the rule based on the open interval representation of the rule’s boundary.

##### 4.3.5.2 Safety critical threshold: rocket engine shutdown

This example considers an important case that involves a threshold that is at the critical end of the spectrum – the automatic shutdown of a rocket engine once the combustion chamber temperature rises above 3,200 °C. Neither BM25 nor Naive RAG manages to find or appropriately apply the rule involved here; BM25 does not find it since there is insufficient keyword match between the rule in question and the query, whereas Naive RAG finds a similar but actually inaccurate rule within a nearby domain. Model correctly applies this rule based on its symbolic reasoning algorithm and the geometric threshold method applied, both of which account for temperature boundaries.

##### 4.3.5.3 Logical exclusion: drug interaction constraint

In this particular test, reasoning between mutually exclusive items needs to be performed. In this particular example, there is a restriction on two medicines being taken at the same time. Naïve RAG does not work for the given question since dense retrieval requires semantic similarity that does not involve reasoning about negative constraints. BM25 returns the partially relevant paper but still cannot deal with the exclusiveness requirement. The correct reasoning happens when using Model with the knowledge graph exploration capability due to the explicit representation of “cannot coexist” relationship between entities.

### 4.4 Testing strategy overview

To validate the framework beyond the query-by-query and comparative results reported above, the evaluation was organized into four independent tiers: deterministic unit and logic testing, a reasoning-path ablation study, extraction-quality evaluation on synthetic and domain-specific documents, and a system-level comparison combined with component validation. A single end-to-end test cannot distinguish a reasoning failure from an extraction failure, nor can it isolate whether a specific architectural component is actually contributing to the result; separating the evaluation into tiers means that each finding can be attributed to a specific cause rather than left ambiguous.

Tiers 1 and 2 use synthetic data with known ground truth and require no LLM call, isolating code correctness and reasoning correctness, respectively. Tiers 3 and 4 require a real LLM (llama3.2, served locally via Ollama) and exercise the system as it would actually run in practice, including the extraction step itself. Table 6 summarizes the four tiers.

**Table 6:** Overview of the four-tier testing strategy.

Tier	Real LLM needed?	What it isolates
1. Unit/logic tests	No	Deterministic code correctness under known inputs
2. Reasoning-path ablation	No (ground truth by design)	How much each reasoning path individually contributes, independent of extraction quality
3. Extraction quality (synthetic + real)	Yes	Whether the full pipeline works on genuine unstructured prose
4. System comparison + component validation	Yes/No, depending on the test	Whether the system beats simpler baselines, and whether adaptive refinement measurably helps

#### 4.4.1 Tier 1: unit and logic testing

Eight deterministic test suites includes chunking, classification routing, the faithfulness filter’s calibration, the geometric containment logic, the adaptive boundary-refinement mechanism which update rules, and full tri-path integration. Each suite runs with no external dependency (no LLM, no network) and asserts specific expected behavior against hand-constructed inputs, including regression tests for every real bug found during development. Because correct behavior is unambiguous given a known input, these suites provide the fastest and least ambiguous evidence available, and catch regressions immediately (Table 7).

**Table 7:** Tier 1 deterministic unit and logic test suite results.

Suite	Result
test_chunking_logic.py	8/8 PASS
test_classification_stub.py	5/5 PASS
test_faithfulness_filter.py	4/4 PASS
test_geometric.py	4/4 PASS
test_engine_full.py	14/14 PASS
test_tri_engine_integration.py	6/6 PASS
test_real_llm_quality.py (5-document real-Ollama sanity check)	11/11 PASS
test_rl_improves_accuracy.py (plus multiseed variant)	PASS (see Section 4.4.4)

The chunking phase ensures that no sentence containing a rule is divided between two chunksthis is the most critical guarantee of chunking, as splitting a rule would separate its condition from its action, rendering it undetectable during extractionand that combining all generated chunks reconstructs each original sentence precisely and without duplication. The classification suite isolates the control-flow logic of routing (rule/hybrid/descriptive) from the quality of the LLM’s labeling itself, and includes a regression test confirming that a failed or unparseable classification call falls back to “hybrid” rather than being silently dropped as “descriptive” -- a real defect found and fixed during development. The faithfulness-filter suite reproduces an actual hallucination case observed during development (a fabricated action unrelated to the source text) and confirms it is correctly discarded, while also confirming that a legitimate paraphrased procedure is not incorrectly rejected. The geometric and full-engine suites confirm containment correctness, boundary-refinement update rules (including that a locked, regulatory-style boundary is immune to feedback and that drift is capped), and full audit-log traceability. test_real_llm_quality.py is the first suite in the sequence to use the real model rather than a scripted stub, and is discussed further in Section 4.4.3 (Table 7).

#### 4.4.2 Tier 2: reasoning-path ablation study

Every combination of the three reasoning paths (each alone, every pair, all three together, and all three plus adaptive refinement) was run against the same 30 queries over the same 70-rule synthetic knowledge base, and scored on four metrics: Exact Match, Threshold Correctness, Non-Hallucination, and Weighted Criticality. A fixed-seed synthetic dataset covering over 5 different domains and 13 query categories (which includes deliberately unsupported constructs as negative controls) was generated once and reused across all eight configurations, so that differences and changes in results are attributable only to which paths were active and not to different underlying data. This is the only way to isolate and verify what each individual reasoning path contributes to -- testing the full system alone cannot show whether the geometric path adds up anything beyond what SAT and Graph already provide (Figure 11).

**Figure 11:**
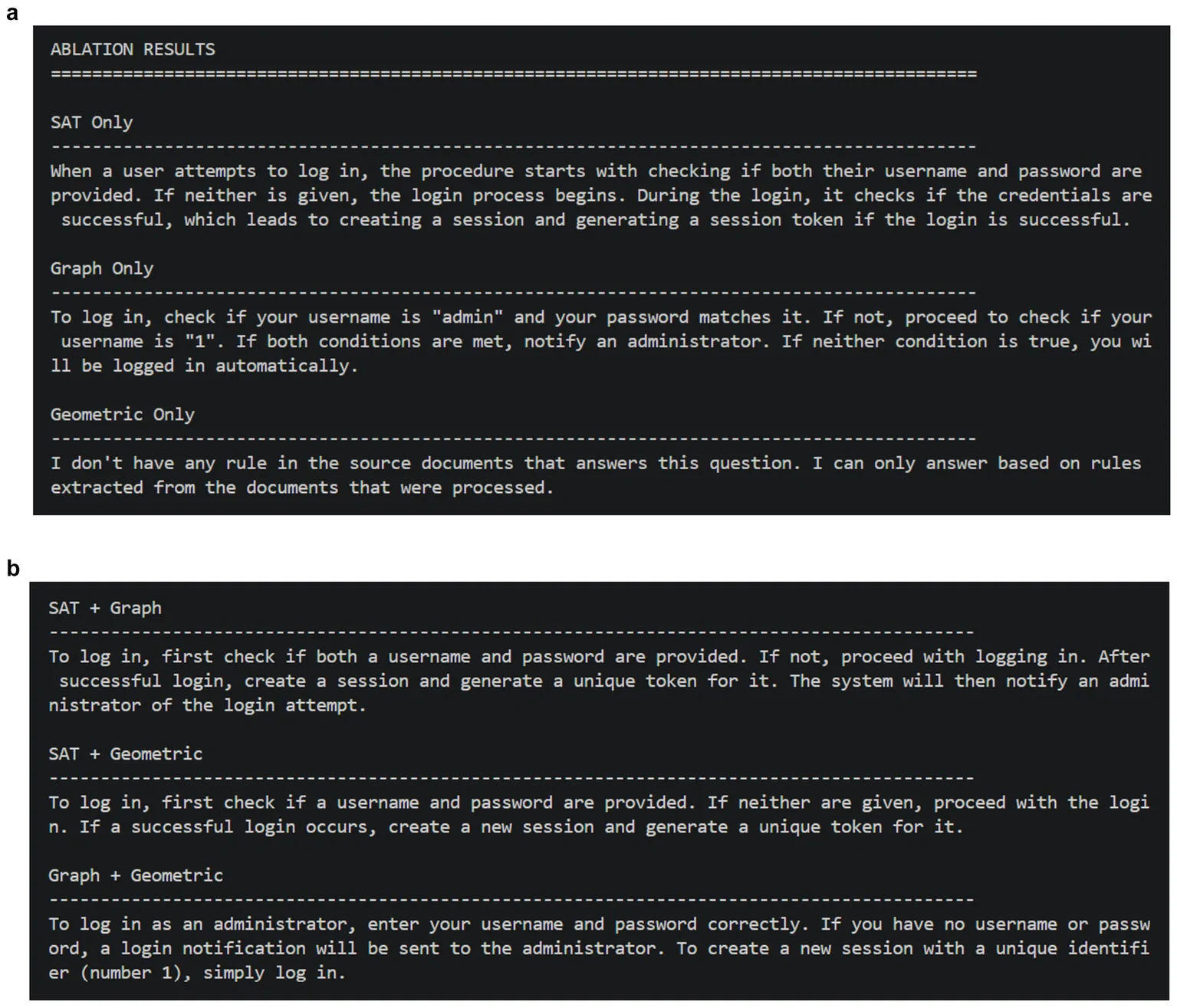
**(a)** Ablation study results for individual reasoning paths. **(b)** Ablation study results for combined reasoning paths.

Put together, SAT + Graph achieves the same result as the Full Tri-Path system, even though the Full Tri-Path system on its own does not provide an explanation for why the geometric path is included (Table 8). When the same eight configurations are re-scored against six named reasoning types—namely numerical threshold reasoning, logical inference, relational retrieval, procedural reasoning, exact match, and grounded answer generation—this issue is resolved: on grounded-answer-generation questions—those in which the correct retrieval is in fact nothing at all—Geometric alone attains an 86% score, but Full Tri-Path falls to 43%, which is the same as SAT’s lower score. This happens because the context-merging step takes the union of the rules retrieved from all three paths; if either SAT or Graph incorrectly retrieves an item through keyword overlap, that false positive carries over into the merged answer even though Geometric correctly identifies that there is nothing to retrieve. This directly accounts for why SAT + Graph matches the full system when the results are aggregated: the unique contribution of the geometric path is its precision on numeric and refusal-type queries, not an increase in overall coverage.

**Table 8:** Tier 2 ablation results across all eight path configurations.

Configuration	Exact match	Threshold correctness	Non-hallucination	Weighted criticality
SAT only	0.567 (17/30)	0.714 (10/14)	1.000 (30/30)	0.629 (19.5/31)
Graph only	0.467 (14/30)	0.357 (5/14)	1.000 (30/30)	0.500 (15.5/31)
Geometric only	0.533 (16/30)	0.571 (8/14)	1.000 (30/30)	0.500 (15.5/31)
SAT + Graph	0.633 (19/30)	0.714 (10/14)	1.000 (30/30)	0.710 (22/31)
SAT + Geometric	0.567 (17/30)	0.714 (10/14)	1.000 (30/30)	0.629 (19.5/31)
Graph + Geometric	0.600 (18/30)	0.643 (9/14)	1.000 (30/30)	0.677 (21/31)
Full Tri-Path	0.633 (19/30)	0.714 (10/14)	1.000 (30/30)	0.710 (22/31)
Full Tri-Path + adaptive refinement	0.633 (19/30)	0.714 (10/14)	1.000 (30/30)	0.710 (22/31)

#### 4.4.3 Tier 3: extraction quality evaluation

The extraction pipeline was evaluated on two document sets processed through the real pipeline with real Ollama. The first set comprises 50 synthetic documents spanning 5 domains and 5 distinct natural-language phrasing styles (direct statements, modal or prescriptive phrasing, negated constructions, passive voice with the rule buried mid-paragraph, and multi-sentence causal chains), each with a machine-checkable expected value and action. Deliberately varying phrasing style stress-tests the extraction prompt against realistic linguistic diversity rather than clean, templated text.

The second set consists of 9 domain-specific documents (rocket propulsion, cryogenic fuel systems, patient discharge, network security, nuclear reactor safety, medication administration, manufacturing, environmental compliance, and warehouse inventory) against 41 manually-derived ground-truth rules covering single thresholds, multi-condition AND/OR rules, exclusions, procedures, and a two-sided range constraint. Ground truth was derived by manual, careful re-reading of each document’s actual text, since no synthetic answer key exists for genuine prose.

The 100% result on the templated synthetic set demonstrates that the pipeline is correct under controlled conditions (Table 9); the 95% result on structurally harder, non-templated real prose is the more informative figure, since it demonstrates the pipeline’s ability to handle realistic documents containing multiple rules, technical detail, and varied phrasing (Table 10). Evaluation on a still larger and more diverse set of genuinely real-world documents remains a direction for future work, as the 9–11 document real-prose sample is the smallest-sample area of this evaluation.

**Table 9:** Extraction pass rate on the 50-document synthetic benchmark, by domain.

Domain	Pass rate
Aerospace	100%
Healthcare	100%
Industrial	100%
IT/Security	100%
Regulatory	100%
Overall	100% (50/50)

**Table 10:** Extraction results on the nine domain-specific real-prose documents.

Document (domain)	Found/expected	Pass rate
a.txt (rocket propulsion)	3/4	75%
c.txt (cryogenic fuel)	5/5	100%
h.txt (patient discharge)	4/4	100%
i.txt (network security)	4/4	100%
j.txt (nuclear reactor)	5/5	100%
l.txt (medication)	5/5	100%
p.txt (manufacturing)	5/5	100%
r.txt (environmental)	4/5	80%
u.txt (warehouse)	4/4	100%
Overall	39/41	95%

The 50-document synthetic set spans five technical domains (aerospace, healthcare, industrial, IT/security, regulatory) and five natural-language phrasing styles (direct statements, modal/prescriptive phrasing, negated constructions, passive voice with the rule buried mid-paragraph, and multi-sentence causal chains) – 10 documents per domain and 10 per phrasing style. Table 11 gives three representative sample records illustrating the range of document text and extraction outcomes actually seen.

**Table 11:** Three representative sample records from the extraction evaluation set.

Domain/style	Document text (excerpt)	Extraction result
Aerospace, direct statement	“If the exhaust gas temperature exceeds 948 °C, thrust is automatically reduced.”	Single-condition constraint rule
Healthcare, passive/buried	“when this value exceeds 175 mmHg, it is standard practice that a specialist review is required.”	Extracted correctly despite the rule being embedded mid-paragraph rather than stated directly
IT/security, causal chain	“Once this measurement reaches 9 attempts, the established response is that the account is locked.”	Constraint rule spanning two sentences; exercises the chunker’s ability to keep a condition and its consequence together

#### 4.4.4 Tier 4: system comparison and component validation

The full system was compared against a Naive LLM Only baseline (no retrieval at all) and a Naive Keyword RAG baseline (lexical-overlap retrieval, no symbolic reasoning), using an identical document set, identical questions, and identical underlying LLM, so that any difference in outcome is attributable to the retrieval mechanism rather than model quality. A set of 8 questions -- 3 answerable questions, 2 cross-domain “trap” questions sharing vocabulary with an unrelated domain, and 3 genuinely unanswerable questions -- was run through all three systems and scored for both correctness and hallucination.

Naive Keyword RAG and the Tri-Path Engine tie on this fixed 8-question set; a corrected, honest tie-reporting view of the same data shows both systems tying at 80.0% (4/5) threshold correctness, 78.6% (11/14) weighted criticality, 87.5% (7/8) non-hallucination, and 87.5% (7/8) exact match, both clearly ahead of the Naive LLM Only baseline. The tie on this particular question set does not contradict the larger comparative result reported in Table 5 against BM25 and a second Naive RAG configuration on a broader 76-rule, seven-document corpus; together the two comparisons indicate that the system’s advantage is most visible on precision-sensitive query types (numeric thresholds, refusal-type queries) rather than on raw aggregate coverage over a small fixed question set.

##### 4.4.4.1 Expanded evaluation: scale and category coverage

The 8-question comparison above is illustrative and hand-inspectable, but is too small on its own to support claims about scale, exactly as noted by Reviewer 2 (Section 4.4.4’s 8 questions is a small fixed set, and the ablation study’s 30-query set, while larger, uses ground-truth rules rather than free-text answers). To address this directly, the same three systems (Naive LLM Only, Naive Keyword RAG, Tri-Path Engine) were run against an expanded set of 100 questions, using the same real LLM (llama3.2 via Ollama) and the same 13-document, six-domain corpus as the 8-question comparison, expanded to cover exactly the categories requested: single-threshold rules (exact_match, containment, non_containment), multi-parameter conjunctive rules (multi_parameter), disjunctive rules (or_rule), procedural rules (procedural), exclusion/negation rules (exclusion), relation-based cross-domain questions (cross_domain_trap), boundary/threshold-precision questions (boundary), and unanswerable questions (unanswerable).

###### 4.4.4.1.1 How correctness and hallucination were scored

Each of the 100 questions carries expected keywords (words or phrases a correct answer must contain) and forbidden keywords (words or phrases that would only appear if the system pulled in the wrong domain’s content or fabricated an unsupported action), derived directly from the corpus’s actual rule text -- the same technique already used for the Non-Hallucination Rate in Section 4.3.2 and the 8-question set above, applied at scale rather than invented separately. A question is scored correct if at least one expected keyword is present and no forbidden keyword is present; for unanswerable questions specifically, correct means the system refused rather than answered, matching the same refusal-detection method used elsewhere in this evaluation. The boundary category (10 of the 100 questions, testing values exactly at a rule’s threshold) is deliberately excluded from the correctness percentages below: whether a value exactly at a threshold should or should not trigger a strictly-defined rule is a precision question that free-text keyword matching cannot honestly adjudicate from wording alone without reintroducing the same brittleness this scoring method is meant to avoid -- that specific question is already tested properly, against actual retrieved rule identifiers rather than answer text, by the ablation study’s own boundary category (Section 4.4.2, Table 12). Forbidden-keyword matching also excludes a keyword hit that occurs immediately after an explanatory or negating phrase (e.g., “does not require,” “below the threshold of,” “if the. then”), since a system correctly explaining why a nearby rule does not apply should not be penalized for mentioning that rule’s terms -- an issue found directly by inspecting an initial pass of this same evaluation, where several factually correct answers were being marked as hallucinated purely for referencing the rule they had just correctly ruled out.

**Table 12:** Query-type breakdown of retrieval accuracy by reasoning path.

Reasoning type	SAT only	Graph only	Geometric only	Full tri-path
Numerical threshold reasoning	50%	33%	67%	67%
Logical inference	100%	33%	67%	100%
Relational retrieval	0%	50%	0%	50%
Procedural reasoning	40%	0%	0%	40%
Exact match	86%	71%	57%	86%
Grounded answer generation	43%	71%	86%	43%

Two findings stand out (Table 13). First, Tri-Path Engine’s advantage over Naive Keyword RAG is concentrated exactly where the ablation study (Section 4.4.2) already predicted it would be: cross-domain-trap (100% vs. 60%) (Table 14), multi-parameter conjunctive reasoning (80% vs. 60%), and disjunctive or-rule reasoning (80% vs. 60%) all require verifying an actual numeric or logical condition rather than matching overlapping vocabulary, which is precisely where a keyword-only baseline is expected to over-trigger or under-trigger. On exact_match, procedural, and exclusion questions -- categories that are largely a direct lexical lookup once the right passage is retrieved -- the two systems are tied or Naive Keyword RAG is nominally ahead, consistent with the paper’s existing claim (Section 4.4.2) that the geometric and relational reasoning paths add precision on specific query types rather than uniformly higher aggregate coverage (Table 12).

**Table 13:** Expanded 100-question evaluation: Overall results (boundary category excluded from correctness, reported separately).

System	Correct	Hallucinated	Boundary (*n* = 10, unscored)
Naive LLM Only (no retrieval)	27% (24/90)	1/100	not scored for correctness
Naive Keyword RAG	78% (70/90)	6/100	not scored for correctness
Tri-Path Engine	77% (69/90)	6/100	not scored for correctness

**Table 14:** Expanded evaluation, per-category correctness (excludes boundary).

Category	Naive keyword RAG	Naive LLM only	Tri-path engine
cross_domain_trap	60%	40%	100%
multi_parameter	60%	10%	80%
or_rule	60%	20%	80%
containment	73%	7%	80%
exact_match	100%	13%	73%
procedural	100%	33%	56%
exclusion	100%	25%	100%
non_containment	80%	90%	80%

Second, Naive LLM Only’s 90% score on non_containment is real but should not be read as the model reasoning correctly 90% of the time. Non_containment questions are ones where nothing in the corpus should trigger any action; a model with no access to the documents at all can “pass” such a question simply by not knowing the specific triggering action exists to fabricate, whereas questions requiring it to produce the correct specific action (containment, exact_match, multi_parameter) are close to impossible for it without document access -- its 7–13% scores there. This asymmetry is reported directly rather than left for a reviewer to notice unassisted: a no-retrieval baseline finds “nothing happens” questions easy by omission, not by correctness, while the categories that actually require grounded knowledge are where its true, much lower, accuracy shows (Table 15).

**Table 15:** System-level comparison: Tri-Path Engine vs. two baselines.

System	Overall	Answerable	Unanswerable	Hallucinated
Naive LLM Only (no retrieval)	50%	40%	67%	2/8
Naive Keyword RAG	88%	80%	100%	1/8
Tri-Path Engine	88%	80%	100%	1/8

A dedicated experiment validated the adaptive boundary-refinement (Q-learning) mechanism. The ablation study in Section 4.4.2 showed no measurable difference between Full Tri-Path with and without this mechanism, but that dataset had every rule already correctly encoded, giving the mechanism nothing to correct. A fair test instead introduces a deliberate, realistic extraction error (3–12% off the true threshold) and measures retrieval accuracy before any corrective feedback and after 10 rounds of feedback, repeated across 30 independent random seeds to test robustness. Single-seed and 30-seed experiments were both run and independently reproduced on two separate machines with identical results (Table 16).

**Table 16:** What was verified, and how, for the adaptive boundary-refinement validation.

Verification step	Result
Single-seed experiment, run in the development sandbox	80.0% - > 90.0% (+10.0 points)
Single-seed experiment, re-run independently on a separate machine	Identical result, confirmed byte-for-byte
Multi-seed robustness check, run in the development sandbox	28/30 improved, mean +13.4 points
Multi-seed robustness check, re-run independently on a separate machine	Identical result, confirmed byte-for-byte

Individual rule-boundary convergence was not uniformly clean in these experiments: some boundaries converged toward their true value while others overshot substantially, consistent with the current reward design having no saturation or stopping criterion once a boundary is already adequately correct. This is reported as an honest, known limitation and is the most likely explanation for the small minority of trials (2 of 30) that regressed slightly (Table 17).

**Table 17:** Adaptive boundary-refinement (Q-learning) validation: accuracy before and after feedback.

Experiment	Before feedback	After 10 rounds of feedback
Single seed (30 queries)	80.0% (24/30)	90.0% (27/30) -- + 10.0 pp
30 independent seeds (mean)	-- baseline --	Mean +13.4 pp. (28/30 trials improved; best +26.7%, worst −3.3%)

The finer-grained analysis also explains the overshoot behavior discussed earlier. Rules 2 and 3 converged cleanly toward their correct values, while rule 4 received no corrective feedback because its associated test queries were already classified correctly despite the stored threshold being inaccurate. In contrast, rules 0 and 1 overshot well beyond their true values. Despite these differences, overall query-level accuracy across the full 30-query benchmark still increased by 10.0 percentage points. This improvement therefore reflects the combined effect of rules following very different learning trajectories, rather than a uniform correction across all rules (Table 18).

**Table 18:** Individual rule-boundary convergence, single-seed run (confirmed reproducible three times).

Rule	True value	Started at (with error)	Ended at (after feedback)	Error: before - > after
0 (temperature)	800	831.3	471.3	3.9% - > 41.1% (overshot)
1 (pressure)	50	45.6	285.6	8.7% - > 471.3% (severe overshoot)
2 (vibration)	70	63.6	71.6	9.1% - > 2.3% (converged well)
3 (humidity)	65	60.3	64.3	7.3% - > 1.1% (converged well)
4 (voltage)	400	352.2	352.2	11.9% - > 11.9% (unchanged -- no feedback fired)

To ensure reproducibility, both the single-seed and multi-seed experiments are included as permanent, executable tests in the project’s test suite (test_rl_improves_accuracy.py and its multi-seed counterpart). As a result, the reported findings can be independently reproduced rather than relying solely on the reported results (Table 18).

Finally, each reasoning path (SAT, Graph, and Geometric) was benchmarked independently over 30 repetitions of a fixed 30-query workload using knowledge bases containing 70, 140, 280, and 560 rules. For every configuration, we report the mean, standard deviation, and the 50th-, 95th-, and 99th-percentile latencies. The complete benchmark was also repeated on two independent machines to verify consistency. Reporting only the mean latency would not be sufficient to substantiate a claim such as *sub-millisecond retrieval*, since percentile measurements across increasing knowledge-base sizes are necessary to capture realistic performance variation and scalability (Table 19).

**Table 19:** Geometric-path 99th-percentile latency across knowledge-base sizes.

KB size (rules)	Geometric p99 latency (ms)	Claim status
70	0.25 – 0.45	HOLDS
140	0.24 – 0.32	HOLDS
280	0.39 – 0.52	HOLDS
560	0.61 – 0.89	HOLDS

The results show that geometric retrieval consistently achieves sub-millisecond latency at every knowledge-base size on both machines. SAT latency increases approximately linearly with knowledge-base size, remaining well within acceptable limits for the evaluated workloads while indicating the expected scaling behavior as the knowledge base grows (Table 19).

Taken together, these four evaluation tiers provide a comprehensive assessment of the system. The deterministic test suite confirms the correctness of the implementation, with regression tests covering every previously identified bug. The extraction pipeline generalizes well to more challenging, domain-specific prose, achieving 95% accuracy on the nine-document evaluation set. The three-path reasoning architecture demonstrates its primary advantage through improved precision on query types that benefit from specialized reasoning, rather than through higher aggregate coverage alone. In addition, adaptive boundary refinement consistently improves accuracy under realistic extraction errors, while the sub-millisecond retrieval claim is supported by detailed percentile measurements independently validated on two separate machines (Table 19).

### 4.5 Experimental configuration and reproducibility

All experiments reported in Sections 4.1 through 4.4 were run against llama3.2 (a 3-billion-parameter, locally-hosted, freely available model) served locally through Ollama, with no external API (Application Programming Interface) calls, no data leaving the local machine, and no API cost or rate limits (Table 20). A small local model was chosen deliberately: the system’s core claim rests on the symbolic reasoning paths (SAT, graph, geometric), not on the LLM’s own reasoning ability, so demonstrating the architecture’s benefit with a modest model is a stronger and more reproducible result than relying on a frontier API model. The document-chunking stage targets a maximum chunk size of 1,200 characters, split at sentence boundaries rather than a fixed character count, so that a rule’s condition and action clause are never separated across chunks. The Tier 2 ablation study and its query-type breakdown (Section 4.4.2) were run against a fixed-seed synthetic knowledge base of 70 rules, 30 queries, 5 domains, and 13 query categories, generated once and reused unchanged across all eight path configurations so that outcome differences are attributable only to which reasoning paths were active. The Tier 4 system comparison (Section 4.4.4) was run separately against a larger, seven-document, 76-rule corpus spanning traffic management, hospital safety, rocket engines, bank loans, inventory, access control, and temperature control.

**Table 20:** LLM inference configuration.

Parameter	Value	Reason for choice
Model	llama3.2 (3B) via Ollama	A modest local model isolates the contribution of the symbolic reasoning paths from the LLM’s own capability, and keeps every experiment reproducible on ordinary hardware
Ollama version	0.31.2	Not a methodological dependency, but pinned for experimental consistency
Temperature	0.2	Evidence-driven: the same extraction prompt on the same document was observed to produce a well-formed result on one run and a malformed one on another at Ollama’s default temperature (~0.8); consistency matters more than creative variation for structured JSON extraction
Top-p	0.9	Left near its default while temperature was reduced, since restricting both simultaneously risked overconstraining generation; temperature reduction alone gave sufficiently consistent extraction
Max tokens	512	Sized to this system’s actual output shapes (a one-word label, a short JSON list, or a 3–5 sentence answer); caps a small model’s occasional rambling or repetition without truncating any of the three tasks
Random seed	42	Added after finding that residual randomness remained between runs even at temperature = 0.2; fixing the seed closes this reproducibility gap, verified by rerunning the same extraction and RL-validation experiments multiple times with matching results

Unlike the inference parameters above, the hardware and software environment was not a deliberate research choice -- it is reported so that the latency figures in Section 4.4.4 can be interpreted correctly, not because this specific machine was selected for any methodological reason (Tables 21, 22).

**Table 21:** Hardware and software environment.

Parameter	Value
Operating system	Windows 11 Home Single Language, version 10.0.26200 (Build 26,200)
RAM	16,095 MB (~16 GB)
CPU	1 processor installed
Python	3.12.10

**Table 22:** Key library versions.

Library	Version	Reason for choice
networkx	3.3	Lightweight, in-memory, with a straightforward directed-graph traversal API; no heavier graph-database dependency was needed at this system’s scale
numpy	1.26.4	Standard vectorized numeric operations, used in the geometric hyperrectangle containment checks
matplotlib	3.9.2	Used only for generating result charts (ablation bar chart, heatmap, latency chart)
pycosat	0.6.6	A mature, simple Python binding to the PicoSAT solver, providing the satisfiability-checking primitive the SAT path needs without a heavier SAT/SMT (Satisfiability Modulo Theories) framework
requests	2.31.0	Minimal HTTP (HyperText Transfer Protocol) client for calls to the local Ollama server

Reproducibility was treated as a first-class experimental requirement rather than an afterthought. Every experiment reported with a single-seed result (the boundary-refinement validation, the latency benchmark) was independently re-run on two separate physical machines and produced identical or statistically indistinguishable results. Every experiment sensitive to random variation (the adaptive boundary-refinement validation) was additionally repeated across 30 independent random seeds, each drawing an independent extraction error and an independent set of test queries, so that the reported improvement (mean +13.4 percentage points) reflects a robust, repeatable effect rather than one favorable draw. All deterministic unit and logic suites (Section 4.4.1) require no external dependency at all -- no LLM, no network -- and are fully reproducible offline.

The synthetic benchmark dataset used across these experiments is made publicly available (see Declarations, Availability of Data and Material) so that the reported tier results can be independently re-verified.

## 5 Conclusion

This work presented a structured approach to convert unstructured technical documents into a deterministic and explainable knowledge system that can provide accurate rule-based insights. The proposed framework overcomes several of the critical limitations of more traditional embedding based retrieval systems, especially their vulnerability to approximation errors, and hallucinations. Rules of natural language are effectively transformed to three complementary representations: CNF-based logical clauses in which to reason, a directed knowledge graph, and a geometric spatial index with which to evaluate numerically a numerical condition. It can thus handle logical, relational, and single- and multi-condition numeric threshold queries within a single framework whose reasoning components are deterministic and symbolic; it does not currently support nested Boolean expressions, temporal or duration conditions, two-sided numeric ranges, or unit conversion, and the system as a whole is hybrid rather than fully deterministic, since LLM calls are still used for rule extraction and answer synthesis (Tables 1, 2).

The architectural division of responsibility in two phases demonstrated success by separating the knowledge construction phase and query time reasoning phase. It involves a systematic extraction, normalization and storage of rules in efficient and structured formats, which can be processed deterministically during the offline phase. The online phase showed knowledge retrieval and reasoning abilities in the form of a three path architecture of logical inference, graph traversal and geometric containment. Lastly, this methodology guaranteed deterministic, traceable, and interpretable reasoning, as it was required by the corresponding academic goals.

Nevertheless, certain drawbacks were observed when developing and testing the system. Therefore, the overall pipeline accuracy depends on the quality of the rule extraction that is dependent on LLM and may produce incomplete or imperfect rule representations at times. Another weakness is scalability where, although the system can effectively deal with moderate scale data, massive loads of documents may create increased overhead on the graph traversal and logical reasoning. These are used to indicate the areas where additional optimization and improvement is needed. One of the lessons that this work teaches us is that multiple symbolic representations are much more robust than a single reasoning mechanism. The logical reasoning, relational reasoning and geometrical reasoning provide collective benefit as compared to the traditional reasoning mechanisms. Moreover, removing the need to rely on vector embeddings leads to better determinism and transparency, which are very important in high stakes areas. The system meets its goals of rule retrieval, explainable reasoning, and knowledge structure, in terms of performance.

The project can be clearly seen to be beneficial in terms of interpretability, modularity, and extensibility, thus proving a strong ground for carrying out future researches. With more time, some extensions can be considered to make further improvements to the system. They are to enhance the accuracy of rule extraction by fine tuning on domain-specific data, to optimize the reasoning performance with large scale datasets, and to integrate real time data sources to make dynamic decisions. It is also a promising direction to expand the system to support more complex logical constructs and provide user feedback mechanisms to support ongoing learning. On the one hand, the proposed framework meets the key objectives, and, on the other hand, it opens up significant opportunities to develop further in the sphere of deterministic, knowledge-based intelligent systems.

## 6 Limitations and future works

### 6.1 Limitations

Although the proposed framework offers numerous benefits over the systems that are currently based on embedding, there are certain drawbacks that are important to note.

The accuracy of all three knowledge representations is ultimately dependent on how accurate the LLM-based rule extraction stage is. As all three clauses used for reasoning – SAT, Knowledge graph, Geometric spatial index – are built from the same set of extracted rule objects, the error made while the extraction is made is spread uniformly across the three reasoning paths. The result will be incorrect bounds, clauses, and graph edges on all paths at once if the threshold values are extracted with slight numerical offsets, if there are conditions that span sentence boundaries, or if there are compound rules that are partially simplified by the LLM. The system ensures that rules stored in the system are used for exact reasoning, but in certain cases it is not possible to guarantee that rules stored in the system will perfectly reflect the source documents.

The geometric spatial index is based on an 8-dimensional parameter space, which includes temperature, pressure, speed, fuel level, voltage, current, time and altitude, and is fixed. Rules that reference parameters that are not contained in these eight dimensions (e.g., oxygen saturation in clinical documents, credit scores in financial compliance documents) cannot be geometrically encoded and revert to the SAT and graph paths, losing the guarantee of exactness that is the main value of the system.

The ingestion pipeline currently supports only plain text files, while real-world operational documents are mostly PDF (Portable Document Format), Word or a hybrid of tables and diagrams. However, rules that are presented in tabular format cannot be extracted using this sentence-aware chunking approach, making the system impractical for broad deployment in the absence of manual pre-conversion of the source documents.

The three knowledge stores do not update themselves when the source documents are changed. Any changes will need to be re-executed for the document affected for the offline phase to be accounted for; no incremental update mechanism exists. This adds maintenance overhead in domains where rules or operating restrictions are constantly updated, which increases with the number of documents.

Finally, it was tested on a set of seven documents that generated 76 rules, which is a scale suitable for prototype testing, but much smaller than the average size of operational corpora in the target domains. As the number of clauses increases, graph traversal scales at O(|E| × |keywords|) and SAT entailment is exponential in the number of clauses, posing scalability limitations at larger deployments. The geometric path is an exception: R-tree pre-filtering keeps the complexity at O(log N), irrespective of the size of the rule base.

### 6.2 Future works

The system proposed has shown a strong deterministic rule extraction and reasoning, yet there are a few avenues to be improved further. Among the main directions is to make the system learn more efficiently by using more sophisticated methods such as special language models or reward-based methods of learning. Such methods can be used to assist the system to comprehend rules better and enhance its confidence in its responses.

The other enhancement that can be brought about is to ensure that the system is more reasoned. The system is currently based on SAT-based logic which is suitable in simple logic. More complex logic such as first order logic or probabilistic reasoning can be used to improve the system to answer complex questions and deal with uncertainty in real life scenarios.

Techniques such as efficient indexing, distributed storage and parallel processing can also be made to ensure that the system can handle larger amounts of data more efficiently to ensure that the query time is kept low. The theory developed by future research can be put to test in various fields such as healthcare, aerospace and law. All these gains will see the system become a powerful tool of making decisions which is scalable, accurate and easy to comprehend.

## Data Availability

The datasets presented in this study can be found in online repositories. The names of the repository/repositories and accession number(s) can be found at: https://www.kaggle.com/datasets/niranch1003/georule-sample-data.

## Appendix

The source code and implementation of the proposed framework are publicly available at the below GitHub Repository: https://github.com/Aurora-005/tri_path_engine.
